# Effect of language proficiency on proactive occulo-motor control among bilinguals

**DOI:** 10.1371/journal.pone.0207904

**Published:** 2018-12-12

**Authors:** Jay Prakash Singh, Bhoomika R. Kar

**Affiliations:** Centre of Behavioural and Cognitive Sciences, University of Allahabad, Allahabad, India; Adam Mickiewicz University, POLAND

## Abstract

We examined the effect of language proficiency on the status and dynamics of proactive inhibitory control in an occulo-motor cued go-no-go task. The first experiment was designed to demonstrate the effect of second language proficiency on proactive inhibitory cost and adjustments in control by evaluating previous trial effects. This was achieved by introducing uncertainty about the upcoming event (go or no-go stimulus). High- and low- proficiency Hindi-English bilingual adults participated in the study. Saccadic latencies and errors were taken as the measures of performance. The results demonstrate a significantly lower proactive inhibitory cost and better up-regulation of proactive control under uncertainty among high- proficiency bilinguals. An analysis based on previous trial effects suggests that high- proficiency bilinguals were found to be better at releasing inhibition and adjustments in control, in an ongoing response activity in the case of uncertainty. To further understand the dynamics of proactive inhibitory control as a function of proficiency, the second experiment was designed to test the default versus temporary state hypothesis of proactive inhibitory control. Certain manipulations were introduced in the cued go-no-go task in order to make the upcoming go or no-go trial difficult to predict, which increased the demands on the implementation and maintenance of proactive control. High- proficiency bilinguals were found to rely on a default state of proactive inhibitory control whereas low- proficiency bilinguals were found to rely on temporary/transient proactive inhibition. Language proficiency, as one of the measures of bilingualism, was found to influence proactive inhibitory control and appears to modulate the dynamics of proactive inhibitory control.

## Introduction

To maintain a behavioral goal, we need to avoid interference from irrelevant information or distracters [1]. Braver [1] described two mechanisms of cognitive control (dual mechanism of cognitive control); one is reactive (inhibitory) and the other is proactive (serves a monitoring function). The proactive mode of control can be activated in an anticipated manner before the occurrence of the conflicting event or in a goal-sustaining manner. Reactive control is activated at the detection of the conflicting event. Morales and colleagues [2,3] who used an AX-CPT task and Prior [4] who employed a task switching paradigm, concluded that both reactive and proactive modes of control work together, towards the cognitive benefits observed in a bilingual population compared to monolinguals. The current study examined the status and mechanisms of proactive inhibitory control in bilinguals as a function of second language proficiency.

There is a strong relationship between cognitive control and language processing [5,6]. Bilinguals were found to outperform monolinguals on several linguistic and nonlinguistic executive control tasks such as the Simon task [7], Flanker task [8], Go/No-Go task [9], Stroop task [10] and Attention Network Task [6] across age including children and older adults [11]. Various factors associated with bilingualism such as the age of language acquisition [12] and second language proficiency interact with the executive control mechanisms among bilinguals [13–16]. Bilingualism might influence different components of control processes as a function of the above-mentioned factors.

Researchers have been debating the locus of cognitive advantage in the bilingual population. Inhibitory [17], monitoring [8] and anticipation [18] accounts have been proposed as explanations for bilingual cognitive control advantage. The continuous practice of the inhibition of non-target language for a bilingual has been one of the major explanations for the cognitive advantage observed among bilinguals over monolinguals [19]. In a recent meta-analysis, Hiltchey and Klein [20] proposed two hypotheses, the bilingual inhibitory control advantage (BICA) and the bilingual executive processing advantage (BEPA) hypothesis. The BICA hypothesis argues for a task general advantage with respect to inhibitory control involved in language selection. The BEPA hypothesis proposes a global cognitive advantage in executive processing. Both hypotheses have gained support from empirical studies [8,21, 22]. In the current study, we expect to find support for the BICA hypothesis given the specific manipulations in the paradigm aiming to look at proactive inhibitory control.

Recently, there has been an upsurge in the literature that offers evidence against bilingual cognitive advantage as a function differences in tasks used/processes examined, low levels of convergent validity across control tasks, sample size, and non-language variables in comparisons between bilinguals and monolinguals [22–24]. An extensive review by Hilchey, Saint-Aubin and Klein [25] highlights the variability in the findings on the cognitive benefits of bilingualism. For instance, Studies that have examined a large sample of bilingual and monolingual children have not found any differences between the two groups on Stroop task and the Attention network task (ANT) [26, 27]. However, the existence of bilingual advantage in young adults and the elderly cannot be ignored [28]. In addition, condition specific effects such as the ability to resolve conflict was found to be related to the level of bilingual experience [29]. Valian [30] reviewed a range of tasks that assessed executive functions and concluded that studies with children and young adults have shown weak and inconsistent effects. Non-language variables mainly socioeconomic status may also co-vary with bilingualism [31].

Overall, the current evidence suggests that bilingual cognitive advantage may not be a robust phenomenon and could be influenced by task specific mechanisms and non-language variables. We could view this as incremental scientific progress toward understanding the relationship between bilingualism and cognitive control. Moreover, studies on bilingual cognitive advantage are primarily based on comparisons of bilinguals and monolinguals, which may or may not generalize to comparisons with respect to proficiency and context based effects on bilingualism.

Two important factors may influence cognitive control processes in the context of proficiency i. e., second language proficiency and the behavioral ecology of a proficient bilingual [32]. Language use is closely related to proficiency particularly in the spoken language domain. According to the behavioral ecology account, bilinguals may use the two languages in single vs dual language contexts, which pose different demands on the control processes. The adaptive control hypothesis of language control suggests that in a single language context, one language is used in one setting and another language is used in another setting and thus frequent switching between languages does not occur [33]. However, dual language context involves the use of both languages with different speakers and some amount of switching between languages. In single and dual language contexts, demand is high on processes such as goal maintenance, monitoring and interference suppression. Second language proficiency is related to such interactional contexts and imposes a demand to adapt the control processes. The bilinguals examined in the current study primarily experienced dual language context (as evident in their self-reported use of L1 and L2 across formal and informal settings) [33].

Language proficiency is a viable measure of bilingualism [34] and modulates language control among bilinguals [35]. Studies on language control and general-purpose cognitive control have shown the effect of language proficiency on interference control, inhibition and anticipation [15, 16, 35]. Proficiency in L2 enhances anticipation, preparation and the language specific selection of words. In this context, the need to facilitate the availability of L2 lexical representations is more than the recruitment of reactive inhibitory control. In other words, the need to exercise proactive control (in order to balance the relative activation of the two languages) is more relevant to support effortless access to lexical representations in high- proficiency bilinguals. The recruitment of language control systems may be generalized to nonlinguistic control tasks.

Research in this area is important in an Indian context where bilingualism is a norm and given the effects of bilingualism on the delayed onset of various subtypes of dementia [36]. Researchers have reported structural plasticity associated with proficiency in a bilingual brain [37] and more specifically the effect of language proficiency on control processes [14, 15, 26, 34, 38–40]. Singh and Mishra [15, 16], Kar and colleagues[34], and Khare and colleagues [14] have reported cognitive control advantage in Hindi-English bilinguals as a function of second language proficiency. Most of the studies in this area have used manual responses. It is likely, that if cognitive benefits were found with manual responses, the advantage should extend to other modes of action, including eye movements, which are known to be closely associated with attentional control [41–44].

To date, only a few studies have examined the effect of bilingualism on occulo-motor control by using cognitive control tasks [15, 45, 46]. Singh and Mishra [15,16] have shown advantage related to monitoring in high- proficiency bilinguals in an occulo-motor Stroop task that involved interference suppression. They did not find an advantage with respect to response inhibition in the double step paradigm [47]. These studies primarily examined the reactive component of cognitive control. However, proactive control mechanisms are required for bilingual language processing.

The current study examined the effect of language proficiency on monitoring and proactive inhibitory control by manipulating the certainty of the nature of the upcoming trial (go or no-go) in a cued go/no-go task. Since, bilingual language processing involves anticipation and proactive mechanisms in order to flexibly adapt to varying linguistic contexts, high- proficiency bilinguals were expected to show better proactive control on a nonlinguistic control task. Two experiments were conducted. The first experiment compared proactive inhibitory control between high- and low- proficiency bilinguals in terms of proactive inhibition cost and adjustments in control by evaluating previous trial effects. We hypothesized that high-proficiency bilinguals would perform better in terms of lower error rates and less inhibitory cost than low-proficiency bilinguals. The second experiment further explored the mechanisms of proactive inhibitory control in order to find the locus of bilingual advantage by testing the temporary state hypothesis and the default state hypothesis of proactive control [48]. According to the temporary state hypothesis, the proactive inhibitory process starts after the predictive/non-predictive cue and proactive inhibitory control is active as soon as there is uncertainty about upcoming go/no-go trial. In this context, inhibitory control is sustained until the uncertainty vanishes. In contrast, the default state hypothesis proposes that proactive inhibitory control is the default state of cognitive control, and can be actively released when an upcoming event becomes predictable. We hypothesized that high- proficiency bilinguals would show evidence for the default state of proactive control whereas low proficient bilinguals would rely on the temporary state of proactive control.

## Experiment 1: Proactive Occulo-motor control among Hindi English Bilinguals

In the first experiment, we examined monitoring and proactive inhibitory control as a function of second language proficiency. We administered 80% of the trials as go trials and 20% as no-go trials coupled with the uncertainty of the occurrence of the upcoming trial type in order to increase the demand on monitoring and proactive control. High- proficiency bilinguals were expected to show better proactive inhibitory control and compared to low- proficiency bilinguals.

### Methodology

#### Ethics statement

The study was approved by the "Institutional Ethics Review Board" University of Allahabad. Written informed consent was obtained from the participants.

#### Participants

A total of 68 student volunteers (41 males and 27 females; mean age (20.54 ± 1.88) from University of Allahabad were first recruited for the study on the basis of following criteria; a) age range of 18–25 years, b) native Hindi speakers with English as their second language; c) at least 7 years of basic education in both languages. All participants reported the use of Hindi at home and use of English in formal settings with respect to spoken language. Language proficiency assessment was conducted for 68 participants using the following screening measures: 1) Language background questionnaire (adapted from) [49] 2) Picture description task [50] and 3) The LexTale test [51].

### Screening measures

#### Language background questionnaire

A language background questionnaire (adapted from [49], see Appendix 1) was administered individually toeach participant to collect information about language use at different ages and communication with family, friends, school, reading, frequency of use of L1 and L2, exposure and self-rated proficiency across languages, language domains and contexts for both Hindi (L1) and English (L2) for reading, writing, speaking, listening and syntax.

#### LexTale test

The LexTale test [51]was administered to assess language proficiency in L2 (English) for both groups. LexTale is a test of English vocabulary knowledge used to assess English proficiency [51]. This test has been previously used as a measure of second language proficiency in bilingualism research on Hindi-English bilingual adults in India [14]. The participants are required to decide if the string of letters presented one at a time is a word or a non-word in English. The proficiency score (in %) based on the LexTale test was generated after the completion of the test. We used a MATLAB script provided on the http://www.lextale.com website to compute the proficiency score. High-proficiency bilinguals obtained a significantly higher score (accuracy) than the low-proficiency bilinguals on the LexTale test and this performance also corresponded with the proficiency ratings on the language background questionnaire.

#### Picture description task

A picture description task was used as an objective measure of spoken language proficiency. In this task, participants were instructed to describe a picture carefully by focusing on the overall theme of the picture along with individual items in the particular picture. There were two pictures and each participant had to describe one picture in L1 (Hindi) and another picture in L2 (English). A grand rubric score (Appendix 1a) was calculated by summing up the scores on the following aspects: a) overall impact and achievement of purpose (whether the participant establishes the main idea), b) organization and techniques (coherence and cohesion with test, method of organization) and c) mechanics (focusing on grammar, pronunciation, presence of pause) [50]. Performance of each participant was rated for the three aspects of discourse by the investigator and was added to provide an overall score. The total score was converted into a percentage score so that it is comparable to the score on LexTale test. Performance on this test was used as a measure of proficiency in L1 and L2. Participants were also matched on L1 proficiency based on their performance, on this task and self-rated proficiency.

#### Results based on the screening measures

The participants who performed the LexTale test and picture description task with more than 80% accuracy were considered highly proficient and those who achieved less than 70% accuracy were considered low-proficiency bilinguals with respect to L2. If a participant showed a discrepancy in the performance on the LexTale test and the picture description (L2) task,i e., if a participant scored above 80% on one measure and below 70% on the other measure, he/she was excluded from the study. Five of 68 participants were excluded from the study for this reason (N = 63). Thus, we categorized 63 participants into high- (N = 33) and low- proficiency (N = 30) groups, with respect to second language proficiency based on their performance on the LexTale test and the picture description task. Performance on the LexTale test was significantly correlated with performance on the picture description task for the second language (English) (*r* (61) = 0.747, *p* = .001). The information included in the subjective report was taken as an additional measure of language proficiency.

Language proficiency measures and context based language use were compared between the two groups (Table 1). Results based on Language background questionnaire suggest that high-proficiency bilinguals exhibited a greater use of L2 and had a higher overall self-rated proficiency in L2 compared to the low-proficiency group. The low-proficiency bilinguals showed greater use of L1, overall self rated proficiency in L1 except for the domain of speaking and understanding. The two groups were comparable with respect to spoken discourse (picture description) in L1, self-rated proficiency in the spoken domain for L1 and age of second language acquisition. Although overall proficiency in L1 was different between the two groups, both groups reported a higher mean rating for their proficiency in L1 (>7 on a 10-point scale). We subtracted self-rated proficiency in L1 from that of L2 for high and low proficient bilinguals. We found a significant difference between the two groups, *t* (61) = -5.21, *p* = .001, which suggests that the difference in self-rated proficiency in L1 and L2 was significantly lower for high-proficiency bilinguals (0.32) compared to low proficient bilinguals (2.12).The use of L2 was reported to be higher in formal settings whereas L1 was used more often in informal settings for both groups.

**Table 1:** Mean comparisons between high and low proficient bilinguals on subjective and objective measures of language proficiency.

Measures	Higher L2 Proficiency Group (N = 33)	Lower L2 Proficiency Group (N = 30)	*pvalue*
Language acquisition and use L1 *	75.03 (13.03)	81.95 (11.3)	.035
Language acquisition and use L2 *	25.58 (13.67)	18.27 (10.94)	.023
Age of second language acquisition	5.27 (2.79)	6.73 (3.27)	0.061
Communication L1 **	52.24 (14.96)	70.64 (16.86)	.001
Communication L2 **	45.85 (12.76)	29.67 (17.07)	.001
L1 informal ***	68.75 (13.97)	81.93 (15.60)	.001
L1 formal ***	30.50 (26.34)	58.65 (26.06)	.001
L2 informal **	28.21 (12.54)	18.06 (15.60)	.006
L2 formal ***	64.89 (24.83)	40.95 (26.24)	.001
Proficiency^1^ L1 (out of 10) *	7.78 (1.36)	8.58 (1.15)	.015
Proficiency^1^ L2 (out of 10) **	8.10 (0.81)	6.46 (1.64)	.001
Reading L1 (out of 10) *	7.57 (2.68)	9.03 (1.58)	.012
Writing L1 (out of 10) **	6.97 (2.81)	8.56 (1.73)	.009
Speaking L1 (out of 10)	9.06 (1.6)	9.43 (0.81)	.256
Reading L2 (out of 10) **	8.84 (1.32)	7.76 (1.69)	.006
Writing L2 (out of 10) **	9.12 (0.89)	7.63 (2.04)	.001
Speaking L2 (out of 10) **	8.39 (1.19)	6.23 (2.28)	.001
Picture description (Hindi)	16.67 (1.63)	15.733 (2.08)	.051
Picture description (English) **	16.21 (1.85)	9.06 (3.56)	.001
LexTale Score (English)**	84.58 (6.67)	60.84 (6.62)	.001

*p < .05

**p < .01

*** *p*< .001

*Note*. Standard deviations are given in parentheses.

^1^Self-rated proficiency (on the scale of 1–10) in reading, writing, listening, and speaking domains.

L1: Hindi; L2: English

There was a significant difference between the performances of the two groups on the LexTale test (*p* = .001), suggesting that with respect to L2 proficiency, the high proficient group was significantly better than the low proficient group. The performances of the two groups were also significantly different on the picture description task in L2 (*p* = .001).

High- proficiency bilinguals performed better on the picture description task in L2 (*p* = .001) with respect to the structure and organization of spoken discourse compared to the low- proficiency group. The two groups were comparable on this task in L1.

In addition, high- and low- proficiency bilinguals were matched with respect to educational level [high-proficiency bilinguals (HP) = 14.84; low-proficiency bilinguals (LP) = 14.83, *t*(61) = .05, *p* = .95]. Educational level was measured in terms of years of education. Socioeconomic status was determined by asking the participants to indicate to which group they belonged by using a 3-point scale, (1 for lower middle class, 2 for middle class, 3 for upper middle class). There was no significant difference in the socio-economic status of the two groups, [HP = 2.27, LP = 2.13, *t*(61) = 1.01, *p* = .31]. Demographic information was collected with the language background questionnaire.

### Paradigm

We employed the cued go/no-go task to investigate proactive inhibitory control among high- and low- proficiency bilinguals. Two types of cues were used: a certain cue (blue circle) and an uncertain cue (red circle). There were three conditions: certain-go trials, uncertain-go trials and uncertain-no-go trials. Slower response times on uncertain go trials compared to certain-go/control trials are a measure of the release of proactive inhibitory control. For optimal performance on uncertain-go trials, the participant is required to withhold the activation of a response and as soon as the target appears for a go response, the participant is required to release the inhibition for a response (saccadic eye movement in the current study). We hypothesized that high-proficiency bilinguals would be faster on uncertain-go trials in comparison to the low-proficiency group and that there would be less of a difference between uncertain and certain go trials in high-proficiency bilinguals than the low-proficiency bilinguals in terms of reduced proactive inhibitory cost.

### Stimuli

Black and white line drawings of living and nonliving pictured objects were used for the experiment. Living and non-living stimuli were taken from the Snodgrass &Vanderwart database [52]. A norming study was conducted in order to select the appropriate pictures for the experiment. A total of 18 students from the University of Allahabad who were proficient in both Hindi and English language volunteered and participated in the norming study. The objects that were labeled living or nonliving by > 80% of the participants were selected for the experiment. Ratings were also obtained from the same 18 participants on the dimensions of familiarity with the pictured object and the frequency of use of the picture name on a 7-point rating scale where a rating of 1 represented very low frequency/familiarity and rating of 7 represented very high frequency/familiarity. The mean frequency rating for selected objects was 5.49 (.81), and familiarity was 5.98 (.60). All the images were of similar size i.e., 300 x 300 pixels. All pictures were presented on a white background.

### Procedure

Eye movements were monitored with aniView X high-speed eye tracking system (Sensomotoric Instruments, Berlin; claimed spatial resolution 0.01°). The stimulus was delivered with Presentation Software (Ver. 16.1) (Neurobehavioral System) on a 19” color monitor, with 1024×768-pixel resolution. The distance between the target display monitor and the observer’s eyes was 65 cm. A chinrest was attached to the system to avoid any kind of unnecessary head movement. Eye movement data were collected with a sampling rate of 1250Hz. If required, a post hoc drift correction was applied after the calibration of the experiment or the beginning of the second part of the experiment on the basis of any systematic deviations in the initial central fixation.

**Fig 1:**
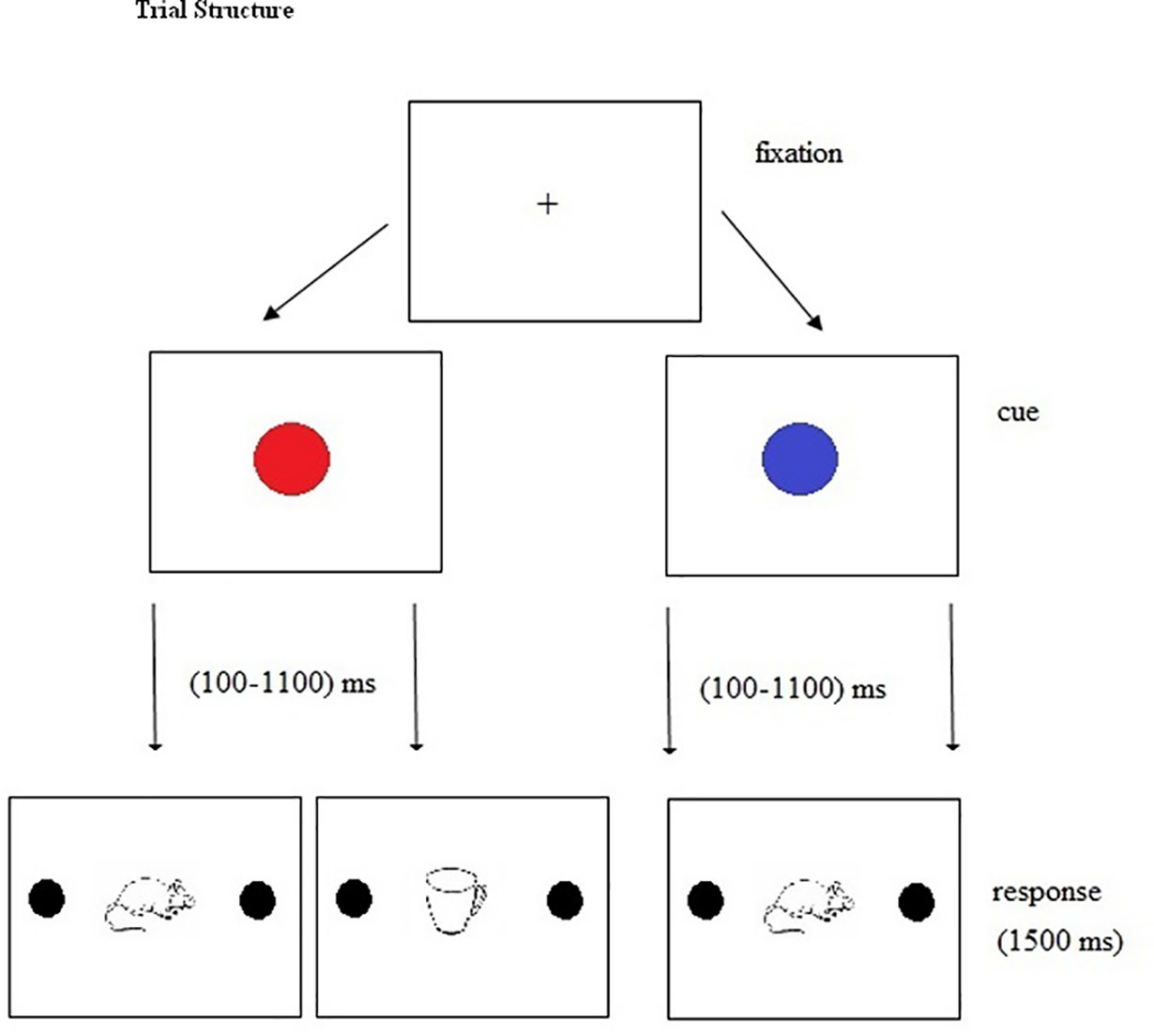
Presents the trial structure and sample stimuli (living and nonliving objects) for the cued go/no-go task. The trial began with a fixation cross, followed by a cue (red or blue circle) for a varied duration of 100-1100ms. The cue was followed by a living or a nonliving object representing a go or a no-go trial with a response window of 1500ms.

The experiment began for each participant with an automatic 13-point calibration. Each trial began with a fixation cross at the center of the screen. The trial would not start until the participant looked at the fixation cross. As participants looked at the fixation cross, a cue in the form of a ‘RED’ or ‘BLUE’ circle appeared on the screen for a duration that ranged between100 ms to 1100 ms. An E-Prime script generated a random number between 100 ms-1100ms for each trial. The blue circle was used as a cue (certain cue) to always predict the upcoming go trial whereas red circle was used as a non-predictive cue and suggested that the upcoming trial may be a ‘go’ or a ‘no-go’ trial, thus requiring greater proactive inhibitory control. Participants were required to fixate at the red/blue circle. Once the red/blue circle disappeared, a pictured object (animate or inanimate) appeared at the center of the screen subtending at 4.2° x 4.2° with two blue circles, one on the left side of the screen (5° from the center) and another on the right side of the screen (5° from the center). Participants were required to move their gaze (go trial) towards the left of the screen on the blue circle, if the object in the picture was animate and they were required to continue to fixate (no-go trial) at the center of the image if the object was inanimate. There were 360 trials in the experiment divided into two blocks of 180 trials each. In the first block, participants were required to move their gaze from the centre of the screen to the left of the screen and in the second block, they were required to move their gaze to the right of the screen for a 'go' trial. The maximum time allowed for responding was 1500ms. Fig 1 presents the trial structure of the first experiment.

The probability of ‘go’ and ‘no-go’ trials was set as 80–20 with 80% trials being go trials and 20% trials being no-go trials. There were 150 trials with certain (‘BLUE’ circle as cue) go trials; 120 uncertain (‘RED’ circle as cue) go trials; 30 uncertain (‘RED’ circle as cue) No-Go trials; and 60 catch trials (30 with a ‘BLUE’ cue and 30 with a ‘RED’ cue). If a participant made an eye movement towards left or right of the central fixation point on a 'no-go' trial, it was considered as an incorrect trial. Similarly, if the participant made an eye movement in the wrong direction, or did not make an eye movement on a go trial, it was considered as an incorrect trial. Two separate scripts were written for go and no-go trials and two different stimulus codes were used to differentiate between the two trial types.

The direction of the eye movement on go trials was counterbalanced. In addition, target type was also counterbalanced across participants. Half of the participants made a response for animate objects (go trials) and half of the participants made a response for inanimate objects (go trials). A tutorial was run for each participant before starting with the experiment. The participants were familiarized with the stimuli (animate and inanimate pictures) used in the experiment and the procedure of the experiment followed by detailed instructions.

## Results

Saccades and fixations were extracted with the standard Be Gaze 3.0 (SMI) thresholds of 40°/s for peak saccade velocity, 8000°/s^2^ for saccade acceleration, a minimum 0.1° initial displacement, a minimum saccade duration of 22 ms, and a minimum fixation duration of 50 ms. First, saccadic latencies were calculated after the onset of the target picture for all the correct go trials. Trials in which saccadic latencies were less than 80ms or more than 1000 ms [15, 53] were excluded in order to filter anticipatory saccades and very slow saccades (1.88%). In addition, mean±3SD a criterion was used to further exclude trials with fast and slow saccadic latencies (1.59%). We calculated the mean response time (saccadic latency) for both certain and uncertain go trials. The overall mean accuracy was 85.82%. The data from 12 (5 high-proficiency, 7 low-proficiency) of 63 participants had to be excluded since their overall accuracy on the go-no-go task was less than 75%. Hence, the data obtained from 51 participants (high- proficiency: N = 28; low- proficiency: N = 23) was subjected to further analysis.

**Fig 2:**
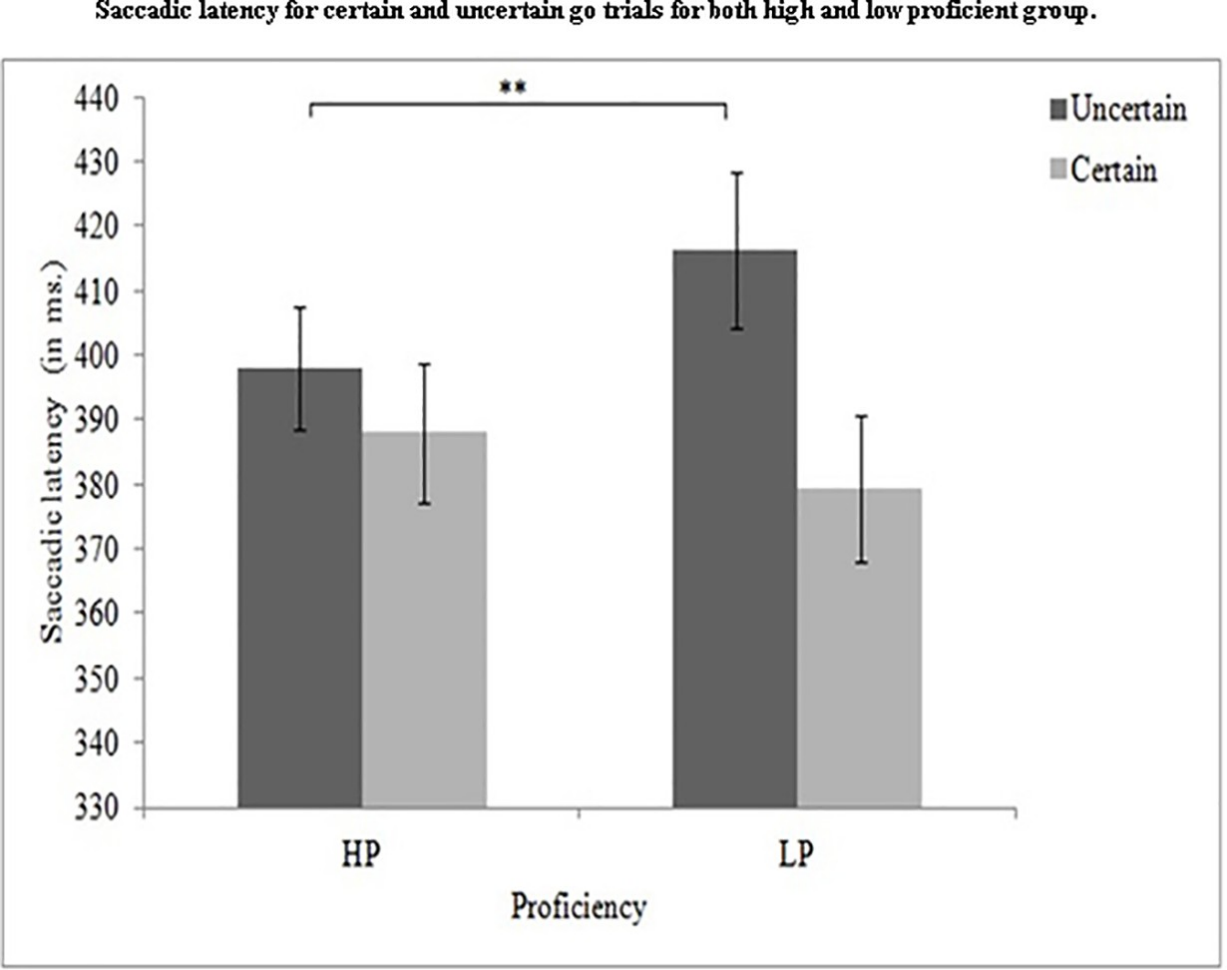
Shows mean comparison of the high- and lo- proficiency bilinguals with respect to the saccadic latencies (in milliseconds) on certain and uncertain go trials in the cued go/no-go task. HP: High-proficiency bilinguals; LP: Low-proficiency bilinguals.

We performed a mixed ANOVA with a 2 (proficiency: high and low) x 2 (cue type: uncertain, certain) design on the mean response time data of 51 participants. There was a significant effect of cue type, *F*(1, 49) = 35.96, *p* = .001,*η*_*p*_*^2^* = .423. The interaction between proficiency and cue type, *F* (1, 49) = 11.93, *p* = .001,*η*_*p*_*^2^* = .196 was also significant. The post hoc analysis with Tukey’s post hoc test, showed significant difference between certain (*M* = 379.40 ms, *SE* = 11.30) and uncertain (*M* = 416.15 ms, *SE* = 12.02) go trials, *t*(49) = 9.45, *p* = .001, in low-proficiency bilinguals but, there was no significant difference between certain (*M* = 388.07 ms, *SE* = 10.77) and uncertain go (*M* = 397.96 ms, *SE* = 9.44) trials for the high-proficiency group, *t*(49) = 2.54, *p* = .28. However, there was a significant difference in saccadic latency for uncertain go trials between high- (*M* = 397.96 ms, *SE* = 9.44) and low- (*M* = 416.15 ms, *SE* = 12.02) proficiency bilinguals, *t*(49) = 4.67, *p* = .009. The two groups were comparable on certain go trials (HP: *M* = 388.07 ms, *SE* = 10.77 and LP: *M* = 379.40 ms, *SE* = 11.30), *t*(49) = 2.23, *p* = .40. Fig 2 presents the mean saccadic latencies of the two groups on certain and uncertain go trials.

**Fig 3:**
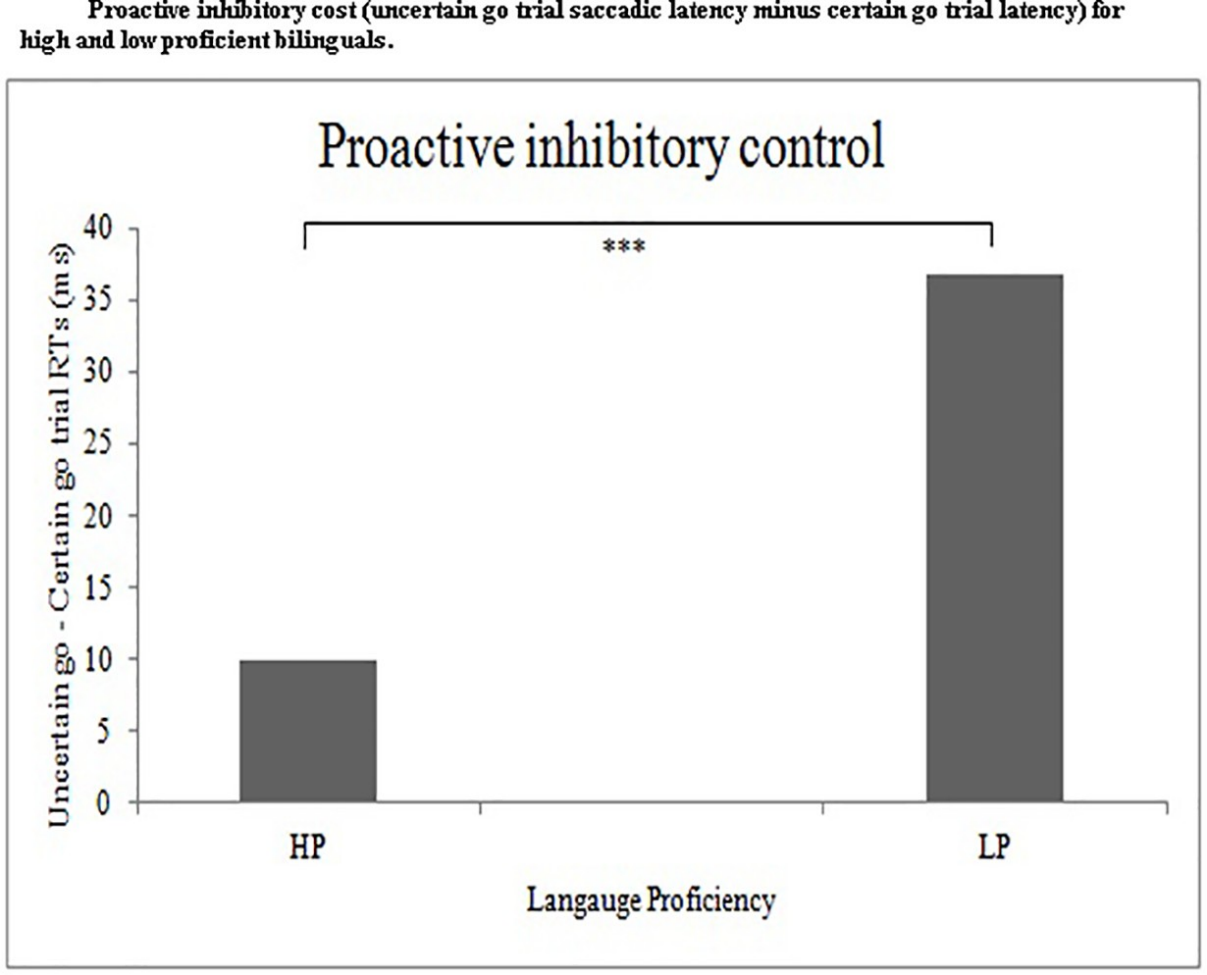
Shows mean comparison of the proactive inhibitory cost (saccadic latency of uncertain go trials minus that of certain go trials) between high- and low- proficiency bilinguals. HP: High-proficiency bilinguals; LP: Low-proficiency bilinguals.

We also computed the inhibition effect by subtracting the saccadic latency for certain go trials from uncertain go trials. We found a significant difference between the low- (36.75 ms) and high- (9.88 ms) proficiency group, *t*(1, 49) = -3.45, *p* = .001, suggesting a reduced inhibitory cost for high- proficiency bilinguals (Fig 3). Differences between certain and uncertain go trial latencies were higher for low-proficiency group than the high-proficiency group, suggesting better non-transient control in high- proficiency bilinguals. Differences between certain go trial and uncertain go trial latencies are known to demonstrate the involvement of proactive inhibitory control [48].

**Fig 4:**
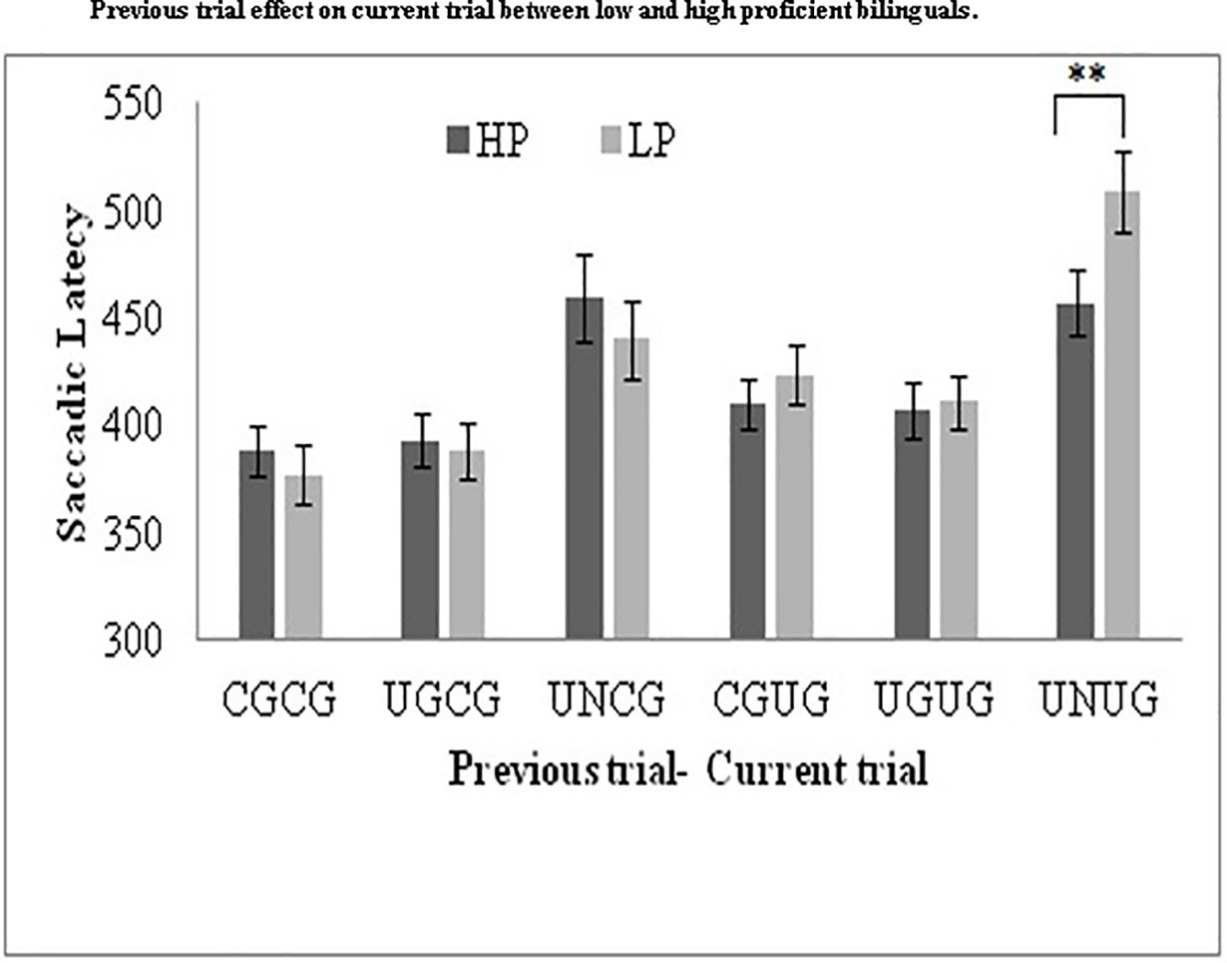
Presenting the effect of previous trial type (certain or uncertain go trial and uncertain no-go trial) on the saccadic latencies of the current trial (certain and uncertain go trials) across six previous-current trial type combinations for high- and low- proficiency bilinguals. HP: High-proficiency bilinguals; LP: Low-proficiency bilinguals; CG: certain-cue go trials; UG: uncertain cue-go trials; UN: uncertain cue-no go trials.

To further investigate the proactive control effects with respect to adjustments in control, we analyzed the data for the previous trial effects (Fig 4). Previous trial effects involve trial-by-trial analysis and provide information about trial sequence effects in controls tasks [39, 54]. Previous trial effects are also a measure of proactive mechanisms in control [55]. We sorted all the trials into 6 categories: uncertain go trial preceded by uncertain go trial; uncertain go trial preceded by uncertain no-go trial; uncertain go trial preceded by certain go trial; certain go trial preceded by uncertain go trials; certain go trial preceded by uncertain no-go trial; and certain go trial preceded by certain go trial. We performed a mixed 2 (proficiency: low and high) x 2 (current trial type) x 3 (previous trial type) ANOVA. There was a significant main effect of current trial type (uncertain go, certain go trial, *F*(1, 49) = 24.99, *p* = .001,*η*_*p*_*^2^* = .338 and previous trial type (uncertain go, uncertain no-go and certain go), *F*(2, 98) = 47.35, *p* = .001, *η*_*p*_*^2^* = .491). The interaction between current trial type and proficiency was significant, *F*(1, 49) = 9.33, *p* = .004, *η*_*p*_*^2^* = .160. There was no significant interaction between current trial type and previous trial type *F*(2, 98) = 1.03,*p* = .35, *η*_*p*_*^2^* = .021.The interaction between proficiency and previous trial type was also not significant, *F*(2, 98) = 0.64, *p* = .52, *η*_*p*_*^2^* = .013. The three way interaction between current trial type, previous trial type and proficiency was significant, *F*(2, 98) = 3.70 *p* = .02, *η*_*p*_*^2^* = .070.

Tukey’s post hoc test for the three-way interaction showed significant difference between the high- (*M* = 457.24 ms, *SE* = 15.85) and low- (*M* = 509.07 ms, *SE* = 18.97) proficiency group, for the current uncertain go trial preceded by the uncertain no-go trial, *t*(98) = 6.03, *p* = .002, which demonstrates that the high-proficiency group had better adjustment in control. In other words, high- proficiency bilinguals were better at releasing inhibition by responding faster on an uncertain go trial when it was preceded by uncertain no-go trial. The remaining comparisons in terms of previous trial effects of certain and uncertain go and no-go trials did not show a significant difference between the two groups (*p*> .05).

Previous trial effects show the effect of the previous uncertain no-go trials, on the latencies of the current certain or uncertain go trials suggesting difficulty in the release of inhibition from the previous demanding trial resulting in greater proactive inhibitory cost among low-proficiency bilinguals. The overall accuracy of both group was comparable, *t*(49) = 0.96, *p* = .34.

Linear mixed effect (LME) analysis was also performed with subjects and items (stimuli) as random effect variables to further explore the mechanisms underlying the recruitment of proactive inhibitory control as a function of proficiency (see results based on LME analysis in S1 File). Results from LME analysis (proficiency x cue type interaction and the effect of proficiency on proactive inhibitory cost) were consistent with those based on ANOVA, particularly the difference between groups for the current uncertain go trials when preceded by uncertain no-go trials (measure of release if inhibition). High proficient bilinguals showed faster release of inhibition with faster latencies on the current uncertain go trial when preceded by the uncertain no-go trial compared to low proficient bilinguals. Subjects and residual factors accounted for the variability in the data.

## Discussion

We examined occulo-motor proactive inhibitory control among high- and low- proficiency Hindi-English bilinguals on a cued go/no-go task. Saccadic latencies were analyzed and the two groups were compared with respect to go trial reaction times (RTs) as a function of cue type (white_cross certain cue and red_cross uncertain cue). Inhibitory control costs as well as previous trial effects were analyzed to investigate proactive control among high- and low- proficiency bilinguals. We hypothesized that high- proficiency bilinguals would be faster on uncertain go trials and would show a reduced proactive inhibitory cost in comparison to the low-proficiency group.

High-proficiency bilinguals were found to be faster than low-proficiency bilinguals on uncertain go trials whereas the two groups were comparable with respect to the latencies on certain go trials. Second, high-proficiency bilinguals showed less inhibitory cost compared to the low-proficiency group. Most importantly, high-proficiency bilinguals showed a greater up-regulation of control than the low-proficiency group, as observed in the analysis based on previous trial effects. Both groups were comparable with respect to the latencies for certain go trials. The high- proficiency bilinguals in the current study showed an advantage with respect to the recruitment of proactive control mechanisms resulting in reduced proactive inhibitory cost. Results based on ANOVA showed faster release of inhibition from a proactively demanding trial (uncertain no-go trial) to a less demanding goal directed trial (certain go trial) in high proficiency bilinguals. In addition the effect of uncertainty was less in the high- proficiency bilinguals than the low- proficiency group resulting in a reduced inhibitory cost. In order to establish enhanced proactive control among bilinguals, we also examined the sustainability of proactive inhibitory control in the second experiment.

## Experiment 2: Effect of language proficiency on the dynamics of proactive control

The cued go/no-go task in the first experiment captured the withholding of a response and the release of proactive inhibition at the onset of target. The second experiment was designed to measure the sustainability and temporal dynamics of proactive inhibitory control using a (slightly) modified version of the cued go/no-go task [48]. Two hypotheses have been proposed to explain how proactive inhibition is implemented in an executive control task, a temporary state hypothesis and a default state hypothesis [48]. We hypothesized that high proficient bilinguals would rely on the default state of proactive control whereas low proficient bilinguals would rely on the temporary state of proactive control. Bilinguals have also shown enhanced attentional control abilities [56] and goal maintenance [57] compared with monolinguals. Second language proficiency also has been associated with enhanced occulomotor control in a conflict-monitoring task [15]. Hence, the locus of bilingual advantage was expected to result in faster release and sustained implementation of proactive inhibitory control among high- proficiency bilinguals.

### Methodology

#### Ethics statement

The study was approved by the "Institutional Ethics Review Board" University of Allahabad. Written informed consent was obtained from the participants.

#### Participants

Sixty-two Hindi-English (Male = 24 and females = 38, mean age = 21.32 years, SD = 2.11) bilingual adults were first recruited for the second experiment on the basis of the basic criteria of age (18–25 years), Hindi as L1 and English as L2 and at least 7 years of education in both languages. Participants were screened via the same measures as discussed in Experiment 1 (see Table 2).

**Table 2:** Mean comparisons between high and low proficient bilinguals on subjective and objective measures of language proficiency.

Measures	Higher L2 Proficiency Group (N = 29)	Lower L2 Proficiency Group (N = 24)	*p value*
Language acquisition and use L1 **	73.56 (13.86)	83.99 (9.35)	.003
Language acquisition and use L2 ***	27.07 (13.77)	15.57 (9.04)	.001
Age of second Language acquisition	5.41 (3.12)	5.87 (2.89)	.58
Proficiency^1^ L1 (out of 10) **	7.88 (1.28)	8.89 (1.01)	.003
Proficiency^1^ L2 (out of 10) ***	7.86 (1.20)	5.81 (1.64)	.001
Communication L1 ***	52.62 (12.65)	70.64 (11.18)	.001
Communication L2 ***	47.60 (12.21)	30.48 (10.95)	.001
L1 use in informal settings*	70.16 (13.06)	79.35 (15.37)	.024
L1 use in formal settings***	31.89 (24.05)	60.08 (19.23)	.001
L2 use in informal settings*	29.60 (11.78)	20.72 (15.63)	.022
L2 use in formal settings***	69.06 (22.49)	42.83 (19.20)	.001
Reading L1 (out of 10) **	7.75 (2.60)	9.45 (1.14)	.005
Writing L1 (out of 10) ***	7.24 (2.24)	9.08 (1.52)	.001
Speaking L1 (out of 10)	9.24 (0.98)	9.58 (0.97)	.21
Reading L2 (out of 10) ***	8.55 (1.63)	6.54 (2.39)	.001
Writing L2 (out of 10) ***	8.62 (1.23)	6.70 (2.42)	.001
Speaking L2 (out of 10) ***	8.27 (1.50)	5.70 (2.34)	.001
Picture description (Hindi)	17.06 (1.55)	16.45 (1.10)	.11
Picture description (English) ***	16.27 (1.94)	8.45 (2.81)	.001
LexTale Score ***	85.92 (6.87)	56.09 (7.02)	.001

*p < .05

**p < .01

***p< .001

*Note*. Standard deviations are given in parentheses.

^1^Proficiency is average of rating (in scale of 1–10) of reading, writing, listening, speaking, syntax and dependence on language.

L1: Hindi; L2: English; participants were identified as high- and low- proficiency based on L2 proficiency.

### Results based on the screening measures

High- and low- proficiency bilinguals were categorized with the same criteria that were used in Experiment 1. Nine out of 62 participants were excluded (N = 53; HP = 29; LP = 24) because there was a discrepancy between the LexTale score and performance on the picture description task with a high score on LexTale and a low score on picture description task or vice versa.

High- proficiency bilinguals showed better use of L2 and overall self-rated proficiency than the low-proficiency group. The low-proficiency group reported greater use of L1, and better overall self-rated proficiency in L1 compared to the high-proficiency group. The two groups were comparable with respect to spoken discourse (picture description) in L1, self-rated proficiency in the spoken domain for L1 and age of second language acquisition. Although overall proficiency in L1 showed a difference between the two groups, both the groups reported a higher mean rating for their proficiency in L1 (>7 on a 10-point scale). The difference between self-rated proficiency in L1 and L2 for high- and low- proficiency bilinguals was significantly different between the high- and low- proficiency groups, *t*(51) *=* -6.04, *p =* .001.The difference between self-rated proficiency in L1 and L2 was significantly lower for high proficient bilinguals (0.02) than for low proficient bilinguals (3.08). Both groups reported a greater use of L1 in informal settings (home family and friends) and L2 was used more often in formal settings. High- proficiency bilinguals performed better on the LexTale test and picture description task in L2 compared to low- proficiency bilinguals. The two groups were matched in their performance on the picture description task in L1 (*p* = .11)

The high- and low- proficiency bilinguals were matched with respect to educational level [HP = 15.51, LP = 15.20, *t* (51) = 1.21, *p* = .22] and socioeconomic status [HP = 2.10, LP = 1.95, *t* (51) = .94, *p* = .34].

#### Stimuli and procedure

Data acquisition and stimulus presentation followed the same procedure as in Experiment 1 using the eye tracking system. The experiment began for each participant with an automatic 13-point calibration. Each trial began with a blank black screen. The trial did not start until the participant looked at the center of the screen. As participants looked at the center of the screen, a pre-cue in the form of a ‘RED’ or a ‘WHITE’ cross appeared at the centre of the screen. The participant was required to keep fixating at the cue until target ‘X’ appeared on the screen. Fifty percent of the times ‘X’ appeared on the left and 50% of the times on the right side of the screen. As soon as the target ‘X’ appeared on the screen, the participant was required to make an eye movement towards the target.

**Fig 5:**
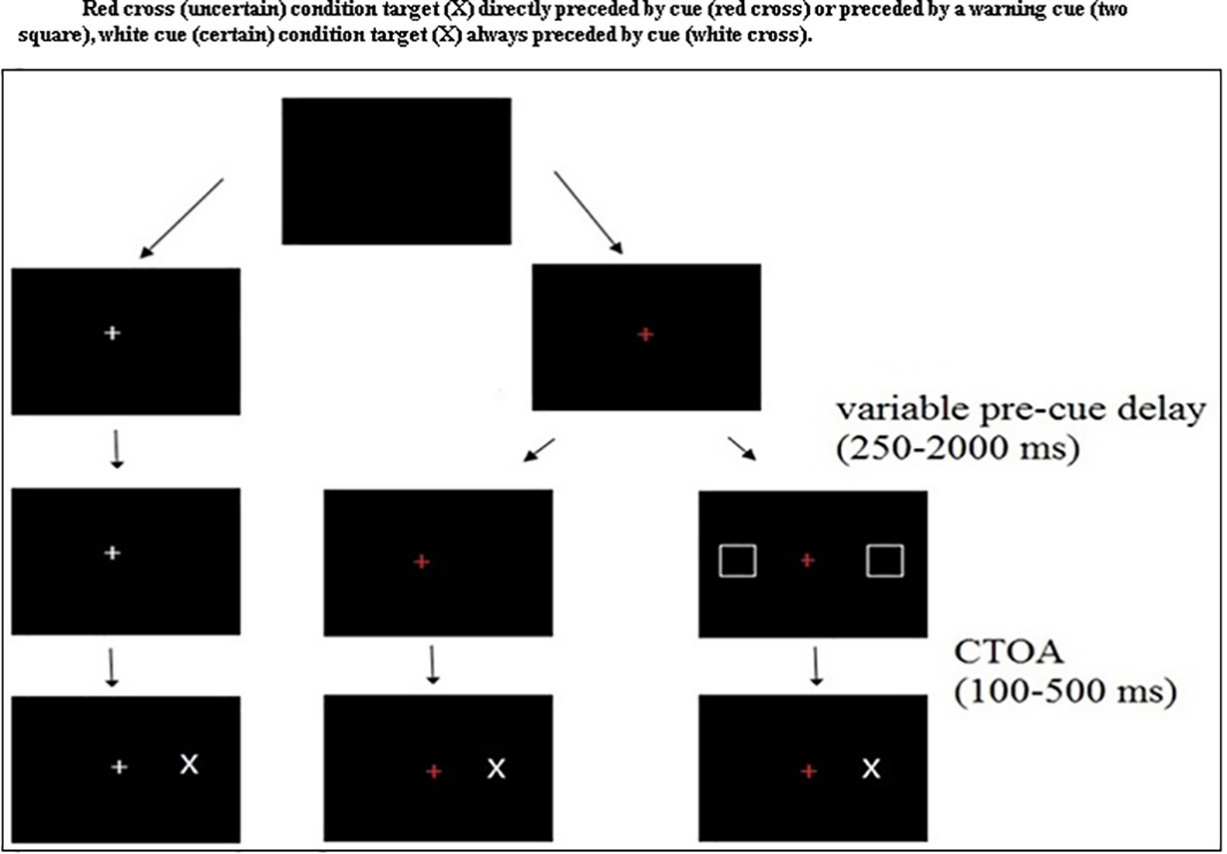
Presents the trial structure and stimuli for the cued go/no-go task with variable pre-cue delay and varying CTOAs between cue and target for the second experiment.

A red cue indicated that in 75% of trials, target would be preceded by a warning cue (two squares one on the left and one on the right side of the fixation cross, 5° from the center) and 25% of trials will not have a warning cue. A white pre-cue indicated that there would be no warning cue before the target. A pre cue delay between the pre-cue (‘Red’, ‘White’ cross) onset and the onset of the warning cue varied between 250 ms and 2000 msand a 250 ms step delay was added to provide time to implement proactive inhibitory control (Fig 5). The pre cue delay was randomly and equally distributed for each trial condition. The warning cue to target asynchrony CTOA (100, 300 and 500ms) was manipulated based on the findings of previous studies [48, 58, 59]. There were a total of 600 trials, 360 trials for the red_cross_with-cue condition (120 for each CTOA, 100, 300 and 500 ms), 120 trials for the uncertain go trial (red_cross_no-cue) and 120 trials for the certain go trials (white_cross_no-cue). Certain go trials were used as a control condition in the experiment. Varying CTOAs were used to examine the recruitment of proactive inhibitory control in terms of default vs temporary state hypothesis. CTOAs were also used to examine release of proactive inhibitory control. Pre cue delay was used as a measure of the sustainability of proactive inhibitory control. A tutorial session was conducted before beginning the experiment to familiarize the participants with the stimuli, trial structure and procedure of the experiment.

#### Data analysis

**Fig 6:**
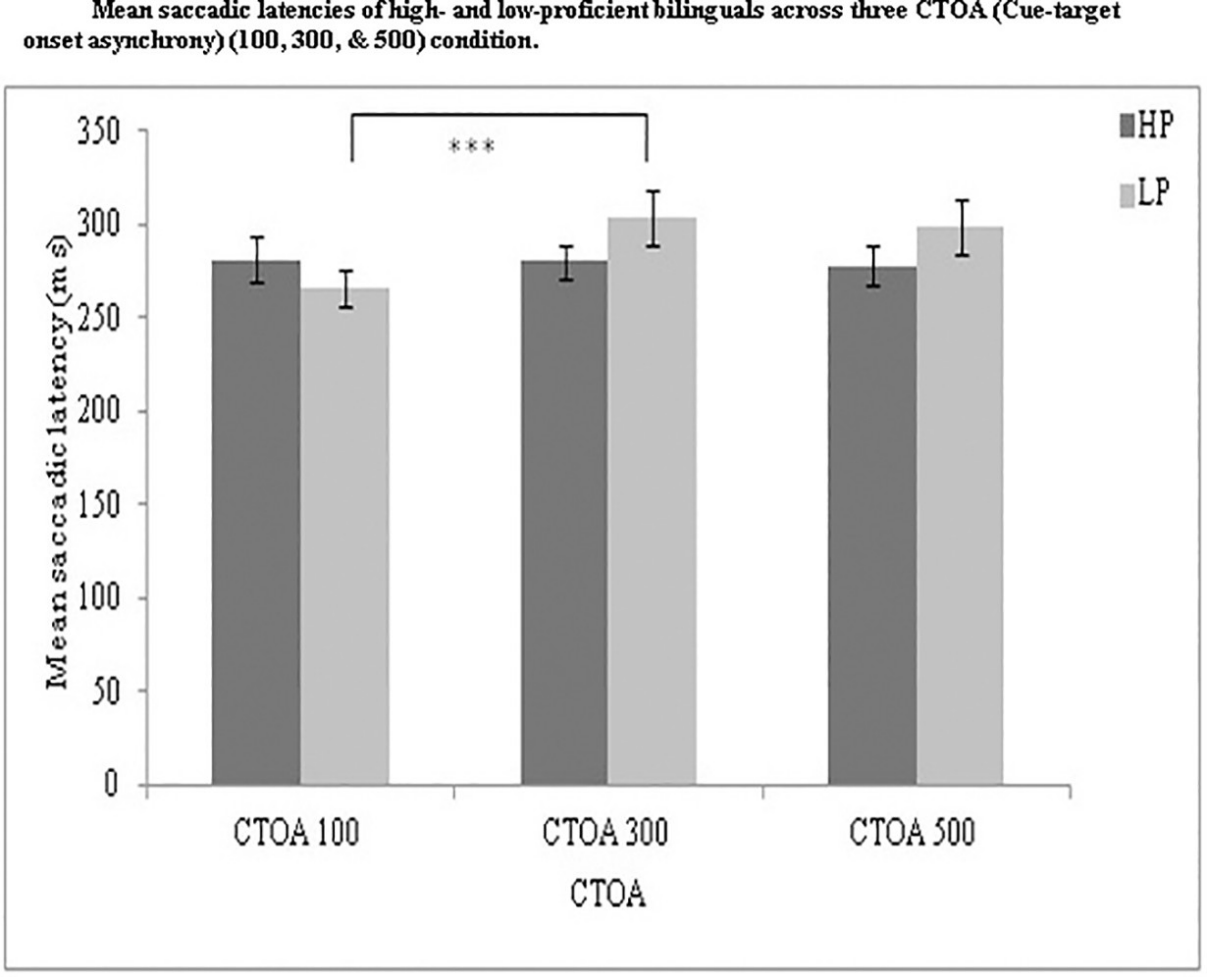
Presents the mean comparisons of the saccadic latencies of high- and low- proficiency bilinguals across three CTOA conditions (100, 300, 500ms). HP: High-proficiency bilinguals; LP: Low-proficiency bilinguals.

Saccades and fixations were extracted following the same procedures as in Experiment 1. We calculated mean saccadic latencies for each participant across all three conditions: uncertain cue without warning signal (red_cross_no-cue), certain cue without warning signal (white_cross_no-cue), and uncertain cue with warning signal (red_cross_with-cue with varying CTOA of 100, 300 and 500ms). We did not exclude any trial from analysis based on accuracy because there was no binary response and generation of a saccade before the target onset would be considered an incorrect trial. The failure to generate a correct saccade suggests a failure of cognitive control (sustainability) in the reactive mode of control [48, 60, 61]. Boulinguez and colleagues [60] suggested that to avoid an erroneous response, proactive control is required to control the response and wait for the target’s appearance, which was evident in the CTOA 100, 300 and 500 conditions (Fig 6).

## Results

### Comparing the two groups across 3 CTOA conditions

Saccadic latencies for the three CTOA conditions were computed for high- and low-proficiency bilinguals (Fig 6). Criaud and colleagues [48] argued that CTOA100 ms is characterized by slower latencies because proactive inhibitory control is still engaged at the onset of the target, whereas CTOA300ms and 500ms is characterized by faster latencies because proactive inhibitory control has already been released by the onset of the target. Thus slower latencies in CTOA 100 ms and comparable for CTOA 300 and 500ms would demonstrate the recruitment of proactive inhibitory control and support the default state hypothesis of proactive control. High- proficiency bilinguals were expected to be slower on CTOA100ms compared to CTOA300 and 500ms conditions.

We performed a 2 (proficiency: HP, LP) x 3 (CTOA: 100,300 and 500) ANOVA and found a significant main effect of CTOA, a faster saccadic latency for 100 ms CTOA, than 300ms, and 500 ms CTOAs, *F*(2, 102) = 4.26, *p* = .017,*η*_*p*_*^2^* = .077 and a significant interaction between proficiency and CTOA, *F*(2, 102) = 5.23, *p* = .007, *η*_*p*_*^2^* = .093. Tukey’s post hoc test showed no significant difference between the saccadic latency for CTOA100 (*M* = 281.09 ms, *SE* = 11.97) and CTOA300 (*M* = 280.12 ms, *SE* = 9.16),*t*(102) = 0.14, *p* = 1.0; or between CTOA300 (*M* = 280.12 ms, *SE* = 9.16) and CTOA500 (*M* = 278.01 ms,*SE* = 11.09), *t*(102) = 0.31, *p* = 1.0 for high-proficiency bilinguals. However, low-proficiency bilinguals showed a significant difference between CTOA100 (*M* = 266.32 ms, *SE* = 9.74) and CTOA300 (*M* = 303.52 ms, *SE* = 14.48),*t*(102) = 5.62, *p* = .001 but not between CTOA300 (*M* = 303.52 ms, *SE* = 14.48) ms and CTOA500 (*M* = 299.01 ms,*SE* = 14.54), *t*(102) = 0.67, *p* = .99. High- proficiency bilinguals showed comparable saccadic latencies across three CTOAs. However, low- proficiency bilinguals were faster in the CTOA100 and their latencies were comparable for the CTOA 300 and 500 conditions suggesting their dependence on the reactive mode of control.

The Linear mixed effect analysis was performed for the two sets of interactions for Experiment 2. Experimental variables were entered as fixed effects and subjects and items were entered as random effects in the model. The proficiency x CTOA analysis showed significantly faster latencies for CTOA 100 than CTOA 300/500 in low- proficiency bilinguals (*M* = 266.32 ms, *SE* = 9.74) compared to high proficiency bilinguals (*M* = 281.09 ms, *SE* = 11.97) (*p* = .02) supporting the temporary state hypothesis of proactive control for low proficiency bilinguals (see S1 File). Subjects and residual factors (not items) as random effects accounted for the variability in data.

### Comparing the two groups across control conditions

**Fig 7:**
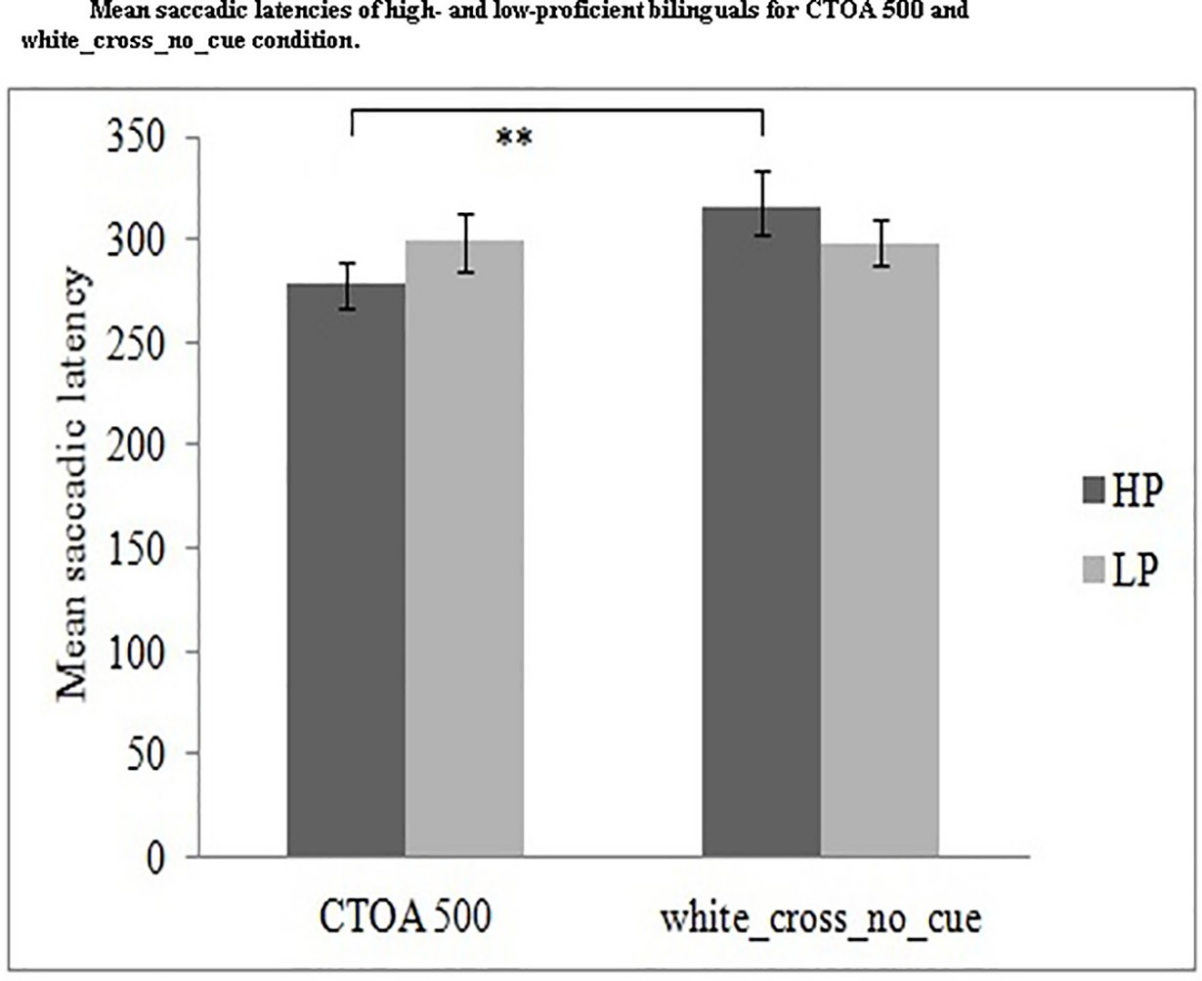
Presents the mean comparisons of saccadic latencies of high and low proficiency bilinguals for CTOA 500ms and white_cross_no_cue (control) condition. HP: High-proficiency bilinguals; LP: Low-proficiency bilinguals.

Criaud and colleagues [48] claimed that certain pre-cue without a warning signal (white_cross_no_cue) as a control condition, is similar to red_cross_with-cue_CTOA 500 condition. The white_cross_no_cue condition does not require proactive inhibitory control. Similarly, the CTOA of 500 ms is sufficient to release the proactive inhibitory control. If proactive inhibitory control is the default state of cognitive control, it would be set up at the onset of the trial and must be released after any stimulus event (white cross or warning cue). We compared white_cross_no_cue with red_cross_500_CTOA conditions, assuming that if a significant difference between the two conditions is observed, it would mean that inhibition is ON as a default state of control and is OFF if there is no significant difference [48]. High proficiency bilinguals were expected to show a significant difference between the two conditions (Fig 7).

We performed a 2(Proficiency: HP, LP) x 2(control conditions: red_cross_with cue_CTOA500, white_cross_no_cue) ANOVA. The main effect of control conditions and the interaction between proficiency and control conditions was not significant, *F*(1, 51) = 3.04, *p* = .08, *η*_*p*_*^2^* = .056 and *F*(1, 51) = 3.14, *p* = .08, *η*_*p*_*^2^* = .058, respectively. Since the interaction effect showed a trend of significance, planned comparisons were performed using paired t test with Bonferroni correction. High- proficiency bilinguals showed a significant difference between certain pre-cue without warning cue (white_cross_no_cue) (*M* = 315.67 ms, *SE* = 13.39) and CTOA500 (*M* = 278.01 ms, *SE* = 11.09), *t*(29) = -2.85, *p* = .008 suggesting that inhibition is ON as a default state of control. However, there was no significant difference between the two conditions in the low-proficiency group.

The LME analysis showed significant main effects and interaction between proficiency and trial type (white_cross_no_cue and CTOA500) (see S1 File). Subjects and residual factors (not items) as random effects accounted for the variability in data. The saccadic latencies of the two trial types (white_cross_no_cue, *M* = 298.77 ms and CTOA500, *M* = 299.08) were comparable for low proficiency bilinguals and the difference between the two trial types (white_cross_no_cue, *M* = 315.67 ms and CTOA500, *M* = 278.01) was found to be greater for high proficiency bilinguals. These results suggest the involvement of default mode of proactive inhibitory control for high proficiency bilinguals.

### Comparing the latencies across pre-cue delays as a measure of sustained proactive inhibitory control

We also manipulated the time duration between the cue and the target as a pre cue delay (250 ms to 2000ms at 250 ms step intervals) to investigate the sustainability of proactive inhibitory control as a function of language proficiency. If one were using a default mode of control, latencies in the short delay (i.e. 250 ms) condition would be slower and would gradually decrease with increasing pre-cue delay. However, if one were using temporary state of control, then response times would be faster and not dependent on the pre-cue delay.

A 2 (proficiency: HP, LP) x 8 (pre-cue delay) ANOVA for CTOA100, CTOA300, CTOA500, red_cross_no_cue, and white_cross_no_cue conditions separately was performed and the results suggest that there is a significant main effect of pre cue delay (*p* < .001) but not a significant interaction with proficiency (*p*>.05). Linear mixed effect analysis also did not show any significant interaction effects between proficiency and pre cue delay across trial types. The results suggest that pre-cue delay affected the response pattern but proficiency did not modulate the role of pre-cue delay on saccadic latencies for any of the experimental or control conditions. The saccadic latencies decreased with increasing pre-cue delay and no differences in latencies were observed after the pre-cue delay of 1250 ms for both groups.

### Accuracy analysis

**Fig 8:**
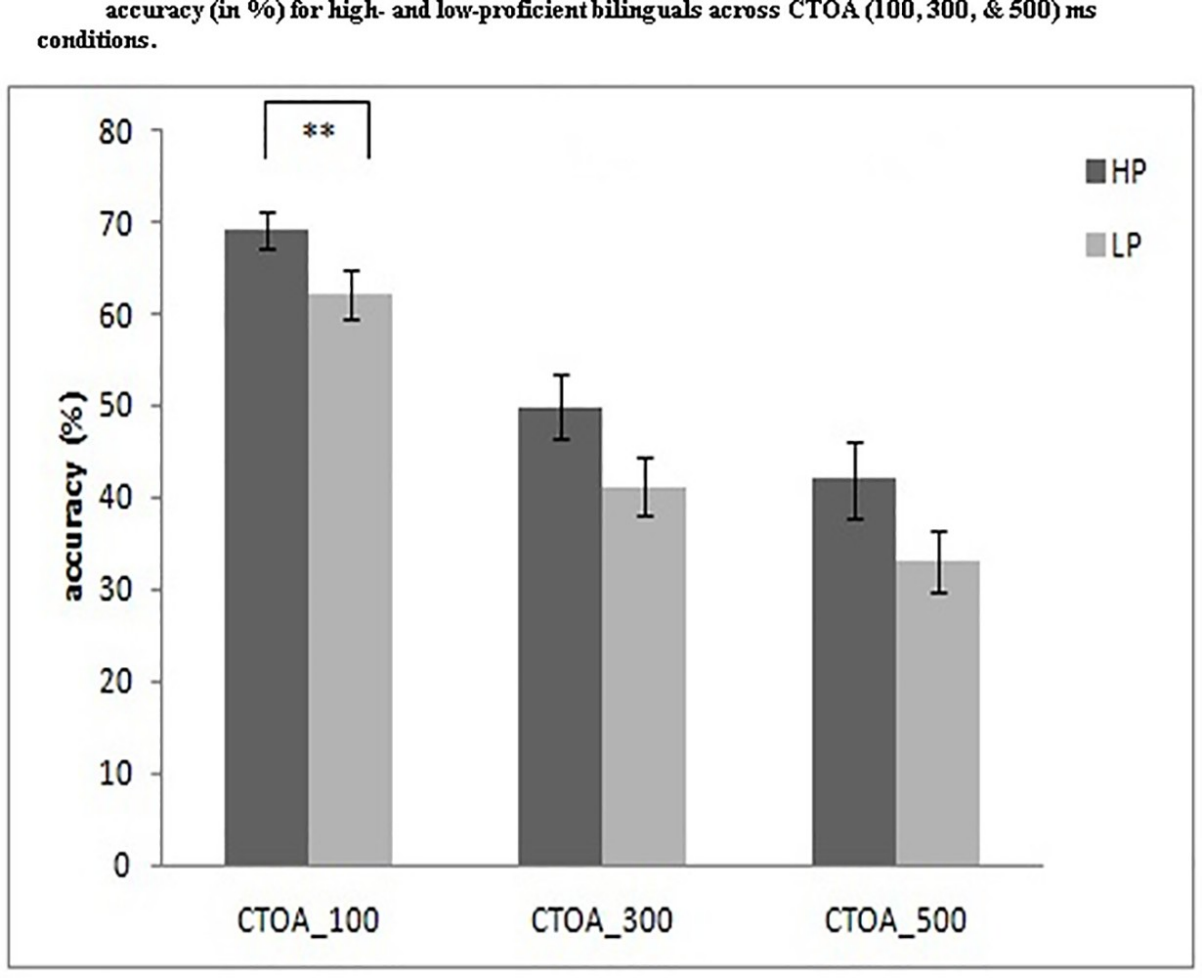
Presents the mean comparisons of high and low proficiency bilinguals with respect to overall accuracy (% of correct trials including both certain and uncertain go trials) across the three CTOA (100, 300, 500 ms) conditions. HP: High-proficiency bilinguals; LP: Low-proficiency bilinguals.

Accuracy data was also analyzed (in %) for CTOA 100, 300 and 500 conditions (Fig 8). We performed a 2 (proficiency: HP, LP) x 3 (CTOA: 100, 300 & 500) ANOVA and the results showed a significant main effect of CTOA,*F*(2, 102) = 127.38, *p* = .001,*η*_*p*_*^2^* = .714 but no interaction between CTOA and proficiency, *F*(2, 102) = .16, *p* = .84, *η*_*p*_*^2^* = .003. The between group difference was significant, *F* (1, 51) = 4.56, *p* = .03,*η*_*p*_*^2^* = .03, which suggests that high-proficiency bilinguals performed with higher accuracy than the low-proficiency group. To evaluate the significant between group differences, we performed independent t-test comparisons and found a significant difference between the two groups for CTOA100 (HP:69.15%, LP:62.18%), *t*(51) = 2.26, *p* = .02, and a trend toward significance for the CTOA300 (HP: 49.88%, LP: 41.20%), *t*(51) = 1.90, *p* = .06, and CTOA500 (HP: 41.95%, LP: 33.04%), *t*(51) = 1.7, *p* = .09, conditions.

Errors on the go no-go task consisted of trials in which participants made a saccade before the appearance of the target, particularly in the context of an uncertain pre-cue without a warning signal (red_cross_no_cue) condition, which requires sustained proactive inhibitory control. High- proficiency bilinguals with a lower error rate showed a better sustainability of proactive inhibitory control. We also calculated the error rates for each pre stimulus delay across all conditions (CTOA: 100, 300 and 500, red_cross_no_cue, and white_cross_no_cue). For each of the five conditions we performed a 2 (HP, LP) x 8 (pre cue delay) ANOVA. The results suggest significant main effects of pre-cue delay and a significant between group differences for CTOA (500ms). The interaction between pre-cue delay and proficiency was not significant. Overall, error rates of high-proficiency bilinguals were lower than low-proficiency bilinguals. To make a correct response in this task, one has to sustain proactive inhibition and make a response only after the onset of the target; if one makes a response before the onset of the target or the warning cue, it is considered an incorrect response [60]. Hence, if high-proficiency bilinguals rely on the default state of control, proactive mechanisms are always active for them, resulting in better accuracy.

### Discussion

The dynamics of proactive inhibitory control were examined in terms of, a) temporary inhibition applied when necessary and b) the default state of the executive control. We hypothesized that high- proficiency bilinguals would rely on the default state of proactive control whereas low- proficiency bilinguals would rely on the temporary state of proactive control. In line with the default state hypothesis, the error rates were expected to be lower across different conditions and mean saccadic latencies were expected to be slower for CTOA100ms compared to CTOA300ms and 500ms. In addition, the latencies were expected to be different between the two control conditions (white_cross_no_cue and red_cross_with-cue_CTOA500) for high- proficiency bilinguals. Saccadic latencies were also expected to decrease across precue delays as a function of sustained recruitment of proactive control in high- proficiency bilinguals.

Experiment 2 revealed mixed results with respect to the default vs. temporary state hypothesis of proactive inhibitory control. Findings in favor of temporary state of control in low- proficiency bilinguals are as follows: a) Faster saccadic latencies for CTOA100 compared to 300ms for low-proficiency bilinguals suggesting a dependence on temporary state of control; b) Overall lower accuracy of low-proficiency bilinguals compared to high- proficiency bilinguals. Findings in favor of default state of control in high- proficiency bilinguals are as follows: a) Slower latencies for CTOA (100ms) compared to low- proficiency bilinguals; b) Significant difference between white_cross_no_cue and red_cross_with-cue_CTOA(500ms) indicating default state of proactive inhibitory control. However, contrary to the expected difference between short (100ms) and long CTOAs (300 and 500ms) we observed comparable latencies across CTOAs (within group effect) for high- proficiency bilinguals, which could also be interpreted as sustained activation of proactive control. The varying precue delays (8 levels) did not show significant effects pertaining to the sustained activation of proactive control in high- proficiency bilinguals.

Overall, low- proficiency bilinguals were found to show reliance on temporary inhibition mechanisms and inefficient recruitment of proactive control. We also find some evidence for the default state of proactive inhibitory control mechanisms in high- proficiency bilinguals. We speculate that in addition to language proficiency, other factors associated with the degree of bilingualism such as L1 proficiency and L2 immersion should also be considered. Moreover, the evaluation of language proficiency as a continuous variable would provide a better measure for its correlation with proactive control mechanisms, given the difficulties in categorizing individuals into two extremes and the observation that language proficiency varies on a continuum. Second, the version of the cued go/no-go task employed in this experiment was complex with many manipulations, resulting in less conclusive effects.

## General discussion

The current study intended to examine the status and dynamics of proactive inhibitory control as a function of language proficiency among Hindi-English bilingual adults. The first experiment showed a significantly reduced proactive inhibitory control cost for high-proficiency bilinguals than low proficiency bilinguals. The second experiment examined the dynamics of proactive inhibitory control and the results are suggestive of temporary/transient proactive mechanisms that underlie the performance of low- proficiency bilinguals. Trends for the presence of default state proactive control were observed among high- proficiency bilinguals.

The reduced proactive inhibitory cost observed among high- proficiency bilinguals in the first experiment is supported by the bilingual inhibitory control hypothesis, which argues for the existence of efficient inhibitory processes in bilinguals compared to monolinguals [20]. However, we did not find a global RT advantage on all types of trials as the two groups were comparable on certain go trials, which goes against the bilingual executive processing advantage hypothesis [20].

In addition to the reduced proactive inhibitory cost, the analysis based on previous trial effects showed faster latencies on uncertain go trials preceded by uncertain no-go trials, which suggests a faster release of inhibition from the previous trial and better monitoring abilities among high-proficiency bilinguals. Monitoring account [56, 60] proposes that a high conflict trial condition affects the upcoming trial in terms of faster reaction time (RTs). Singh and Mishra [47], found larger post-stop slowing in high- proficiency Hindi-English bilinguals in a visually guided redirect (VGR) and memory guided task (MGR), supporting the monitoring account of cognitive control. Similarly, Colzato and colleagues [57] showed a larger attention blink cost for bilinguals supporting monitoring account. It is evident that proficiency modulates cognitive control [40, 62, 63] and the occulo-motor control [15]. One recent study on Hindi-English bilingual adults has also demonstrated an advantage with respect to anticipatory occulo-motor control [45]. The current study demonstrates that proficiency modulates proactive inhibitory control in terms of its implementation as well as the dynamics of proactive inhibitory control.

Since proficiency is a more variable measure of bilingualism, the effects with respect to the different modes of control may manifest in a subtle manner. For instance, we observed better recruitment of control mechanisms in high- proficiency bilinguals under high monitoring conditions such as go or no-go trials with an uncertain cue. In case of the uncertain go/no-go trials, preparedness and monitoring of the upcoming target is required for optimal performance. Low-proficiency bilinguals may also recruit control mechanisms in low or high monitoring conditions but with greater cost in terms of slower latencies and low accuracy, as observed in the current study. Low-proficiency bilinguals were also found to be slower in releasing proactive inhibition.

The second experiment allowed us to demonstrate the locus of advantage related to proactive inhibitory control. The results show some trends in support of the default state of proactive inhibitory control in high- proficiency bilinguals. Performance of the high- proficiency bilinguals shows that triggered by the pre-cue, the proactive control mechanisms were found to be active at target occurrence and inhibition was always ON which is a measure of default state of proactive control. These findings are also supported by the results of linear mixed effect analysis. Better accuracy in case of high-proficiency bilinguals is also an evidence for superior proactive control since the sustained activation of inhibition mechanisms is required particularly in case of the absence of a warning signal following an uncertain cue. Low accuracy observed among low- proficiency bilinguals is suggestive of a failure to inhibit and greater dependence on reactive or temporary/transient inhibition mechanisms. Moreover, we have not found an overall advantage for high- proficiency bilinguals as described in [6] and [15] but we found more subtle points of differences between the high- and low- proficiency bilinguals in a complex cued go-no-go paradigm rather than a global RT advantage.

The subtle or less specific effects of proficiency on proactive inhibitory control mechanisms could also be attributed to the context-based effects of bilingualism. The behavioral ecology account of bilingualism suggests that different contexts influence the recruitment of control mechanisms [32]. Communities that engage less in code switching because their language use may vary across contexts i. e., formal vs informal settings, may show different yet more stable effects related to preparation, anticipation, and monitoring and variable effects on conflict resolution and inhibition as a function of language proficiency. Hence, the subtle effects related to the interaction between proficiency and experimental variables, observed in experiment 2 could be attributed to the language related tradeoffs observed between L1 and L2 use between the high- and low- proficiency bilinguals. High- proficiency bilinguals reported greater use of L2 (English) than L1 (Hindi). However, the use of L2 is more prominent in formal settings whereas use of L1 is more prominent in informal settings for both high- and low- proficiency bilinguals. Such tradeoffs may exist in the population particularly when proficiency is the measure of bilingualism. In such behavioral ecologies, code switching is expected to occur less often, which may dilute the effect of proficiency on control processes. Future studies need to investigate the explicit role of language use across contexts and settings in predicting the proficiency levels as well as the adaptive response to control mechanisms.

The current study is the first to demonstrate that second language proficiency modulates proactive inhibitory control (resulting in reduced inhibitory cost). High- proficiency bilinguals were found to be better at applying and releasing proactive inhibitory control. In addition high- proficiency bilinguals in the current study showed trends for a greater reliance on the default state of proactive control, which is always active regardless of the time to recruit proactive mechanisms. In contrast, low-proficiency bilinguals were found to show greater reliance on temporary/transient inhibition mechanisms. The use of the two languages in single- and dual- language contexts poses greater demands on inhibition and goal maintenance and may result in more efficient proactive inhibitory control mechanisms in high- proficiency bilinguals. The behavioral ecological perspective of bilingualism needs to be further explored in the context of the effect of language proficiency on proactive control mechanisms.

## Supporting information

S1 Appendix (DOCX)

S2 Appendix (DOCX)

S1 File (DOCX)
