# Supplementary material for: Effect of language proficiency on proactive occulo-motor control among bilinguals
Source: PLoS One. 2018 Dec 12;13(12):e0207904. doi: 10.1371/journal.pone.0207904 (PMC6291103; doi:10.1371/journal.pone.0207904)
Supplement: S1 Appendix — (DOCX) [file pone.0207904.s001.docx]

**S1 APPENDIX**

# Language Background Questionnaire

Name: Male/Female: Age:

Mother tongue (L1): Second language (L2): Other languages:

# Hindi language acquisition and use

Use of Hindi at different ages (% Time)

| Age | Percentage of time you speak Hindi |
| --- | --- |
| 0 – 3 years |  |
| 3 – 6 Y. |  |
| 6 – 10 Y. |  |
| 10 – 13 Y. |  |
| 13 – 15 Y. |  |
| 15 – 18 Y. |  |
| 18 Y. - |  |

# English language acquisition and use

Use of English at different ages (% Time)

| Age | Percentage of time you speak English |
| --- | --- |
| 0 – 3 years |  |
| 3 – 6 Y. |  |
| 6 – 10 Y. |  |
| 10 – 13 Y. |  |
| 13 – 15 Y. |  |
| 15 – 18 Y. |  |
| 18 Y. - |  |

Age of acquisition of Second Language (English):

Where did you learn English? (Family, School, Exchange programs, Travel, Work): How many years have you spoken Hindi?:

How many years have you spoken English?: How long have you lived in India?:

Have you ever lived in an English speaking country, e.g., England, America? If yes, when and for how long?:

# Communication with others (Percentage of time in each language):

|  | Hindi | English |
| --- | --- | --- |
| Family (Mother, Father, Grandparents) |  |  |
| Friends |  |  |
| Partner |  |  |
| Work (Boss/Colleagues) |  |  |
| School (Teachers/ Profs/ Classmates) |  |  |
| Free time |  |  |
| TV/ Radio |  |  |
| Reading (Magazines/Books) |  |  |
| Total |  |  |

**Evaluation of Language proficiency in different tasks: Hindi**

Reading (1 = really difficult to understand magazines in Hindi; 10 = very easy)

1 2 3 4 5 6 7 8 9 10

Writing (1 = really difficult to write in Hindi; 10 = really easy)

1 2 3 4 5 6 7 8 9 10

Listening (1 = really difficult to understand spoken Hindi (e.g.,on the telephone); 10 = really easy) 1 2 3 4 5 6 7 8 9 10

Speaking (1 = really difficult to formulate sentences in a conversation in Hindi; 10 = really easy) 1 2 3 4 5 6 7 8 9 10

Syntax (1 = really difficult to decide if a Hindi sentence is grammatically correct; 10 = really easy to detect grammatical mistakes)

1 2 3 4 5 6 7 8 9 10

Dependence on language (1 = I think in English; 10 = I think in Hindi)

1 2 3 4 5 6 7 8 9 10

Dependence on language II: During a conversation, do you need to mentally translate a Hindi sentence to English to understand it? (1 = I always translate Hindi into English to ease understanding; 10 = I never translate Hindi into English)

1 2 3 4 5 6 7 8 9 10

# English

Reading (1 = really difficult to understand magazines in English; 10 = very easy)

1 2 3 4 5 6 7 8 9 10

Writing (1 = really difficult to write in English; 10 = really easy)

1 2 3 4 5 6 7 8 9 10

Listening (1 = really difficult to understand spoken English (e.g., on the telephone); 10 = really easy) 1 2 3 4 5 6 7 8 9 10

Speaking (1 = really difficult to formulate sentences in a conversation in English; 10 = really easy) 1 2 3 4 5 6 7 8 9 10

Syntax (1 = really difficult to decide if an English sentence is grammatically correct; 10 = really easy to detect grammatical mistakes)

1 2 3 4 5 6 7 8 9 10

Dependence on language (1 = I think in Hindi; 10 = I think in English)

1 2 3 4 5 6 7 8 9 10

Dependence on language II: During a conversation, do you need to mentally translate an English sentence to Hindi to understand it? (1 = I always translate English into Hindi to ease understanding; 10 = I never translate English into Hindi)

1 2 3 4 5 6 7 8 9 10

# Switching between languages

**Think about a conversation between you and another person who also speaks both Hindi and English**

Frequency of switching between languageswhen there either is not an English word for something or you do not know the English word for something(English to Hindi) (1 = when I speak English I never use Hindi words in the same sentence; 10 = when I speak English, I always use Hindi words in the same sentence)

1 2 3 4 5 6 7 8 9 10

Frequency of switching between languages when you know the English word for something(English to Hindi) II (1 = when I speak English I never use Hindi words in the same sentence; 10 = when I speak English, I always use Hindi words in the same sentence)

1 2 3 4 5 6 7 8 9 10

Frequency of switching between languages when there either is not a Hindi word for something or you do not know the Hindi word for something(Hindi to English) (1 = when I speak Hindi I never use

English words in the same sentence; 10 = when I speak Hindi, I always use English words in the same sentence)

1 2 3 4 5 6 7 8 9 10

Frequency of switching between languages when you know the Hindi word for something (Hindi to English) (1 = when I speak Hindi I never use English words in the same sentence; 10 = when I speak Hindi, I always use English words in the same sentence)

1 2 3 4 5 6 7 8 9 10

Switching between languages (1 = really difficult to switch between languages in a conversation; 10 = really easy to switch between languages)

1 2 3 4 5 6 7 8 9 10
