# Supplementary material for: Effect of language proficiency on proactive occulo-motor control among bilinguals
Source: PLoS One. 2018 Dec 12;13(12):e0207904. doi: 10.1371/journal.pone.0207904 (PMC6291103; doi:10.1371/journal.pone.0207904)
Supplement: S2 Appendix — (DOCX) [file pone.0207904.s002.docx]

**S2 Appendix**

**Rubric for Scoring the Picture Description Task**

| Strong 3 points | Average 2 points | Weak 1 point |
| --- | --- | --- |
| 1. **Overall impact and achievement of purpose** |  |  |
| 3 Presents a vivid, memorable picture, place or things | 2 presents a clear picture of a person, place, or thing | 1 presents an unclear or confusion or confusing picture of a person, place and thing |
| 3 Established a dominant, or main, impression of the picture | 2 focuses on important characteristic[s] of the picture | 1 presents an unfocused array of characteristics of the picture |
| 3 Conveys a clear sense of purpose | 2 suggests the speakers purpose | 1 unclear or inadequate indication of speakers’ purpose |
| 1. **Organization and techniques** |  |  |
| 3 uses a clear, consistent method of organization of event | 2 Method of organization is usually clear and consistent | 1 method of organization is difficult to identify or follow |
| 3 coherence and cohesion demonstrated through some appropriate use of devices (transition, pronoun, casual linkage, etc) | 2 coherence and cohesion (sentence to sentence) evident, may depend on holistic structure, most transitions are appropriate | 1 evidence of coherence may depend on sequence. If present, transitions may be simplistic or even redundant |
| 1. **Mechanics** |  |  |
| 3 very few, if any errors in grammar, pronunciation and presence of few pauses (filled and unfilled) | 2 small number of errors in grammar, pronunciation and presence of indefinable pauses (filled and unfilled) | 1 numerous errors in grammar, pronunciation and presence of pause (filled or unfilled) |
