# Supplementary material for: Effect of language proficiency on proactive occulo-motor control among bilinguals
Source: PLoS One. 2018 Dec 12;13(12):e0207904. doi: 10.1371/journal.pone.0207904 (PMC6291103; doi:10.1371/journal.pone.0207904)
Supplement: S1 File — (DOCX) [file pone.0207904.s003.docx]

**Linear Mixed effect analysis with Subjects and Items (Stimuli) as random factors**

**Experiment 1:**

R (R Core Team, 2011) and lme4 (Bates, Maechler, Bolker, & Walker, 2015) were used to perform the linear mixed effects analysis to examine the relationship between saccadic latency and second language proficiency. The analysis was performed as discussed by Barr et al (2013) & Jaeger (2009) including by subject and by-item intercepts and by-item slopes as random factors appropriate for a mixed design with both between subject and within subject factors. As fixed effects, proficiency (low, high) and cue type (certain, uncertain) (with interaction term) were entered into the model for the first set of analysis. As random effects, we had intercepts by subject and item, and slope by item (stimuli: living and non-living objects) for the interaction of group and cue type. Visual inspection of residual plots did not reveal any obvious deviations from normality. The *p*-values were obtained by likelihood ratio tests of the full model with the effect in question (Proficiency x cue type) against the model without the effect in question (base model taking proficiency as the fixed factor).

**Saccadic latency: Proficiency x Cue type**

Model1 <- lmer(RT ~ Group + Cue + (Group*Cue) + (1 | Sub) + (Group * Cue | Item) ,data, REML=FALSE)

Table 1. Linear mixed effect models for comparing the saccadic latencies of high- and low- proficiency bilinguals as a function of cue type (certain go, uncertain go) on a cued go/no-go task

Random effects:

Groups Name Variance Std.Dev. Corr

Subject (Intercept) 2448.26 49.47

Item (Intercept) 6.05 2.46

GroupLP 8.64 2.93 1.00

CueU_Go 0.15 0.38 1.00 1.00

GroupLP: CueU_Go 0.38 0.61 1.00 1.00 1.00

Residual 366.06 19.13

Fixed effects:

Estimate Std. Error t value

(Intercept) 388.04 10.03 38.68

GroupLP -8.79 15.07 -0.58

CueU_Go 9.81 5.16 1.90

GroupLP:CueU_Go 26.87 7.63 3.52

*Note*. LP: Low-proficiency; U_Go: Uncertain Go

Results show that the difference (26.87 ms) between uncertain go and certain go trials is different between low and high proficient bilingual groups and a higher intercept value (388.07), which suggest that difference between both conditions is increasing for high proficient bilinguals. Results showed absence of group effect; however proficiency affected performance as a function of cue type for low proficient bilinguals. Intercept is non-zero, which is a significant value. Figure 1 shows that the median line for saccadic latency is higher for low proficient group in uncertain go condition compared to certain go condition. With proficiency and cue type as a fixed factor, variability for random factors is due to subjects, and variability for unexplained factors is very low as compared to subject and variability due to item for proficiency and cue type is lesser in comparison to subject variable.

Second language proficiency affected saccadic latency (χ^2^(11) = 32.84, *p*=0.001), increasing it by about 26.87 ± 7.62 (standard errors).


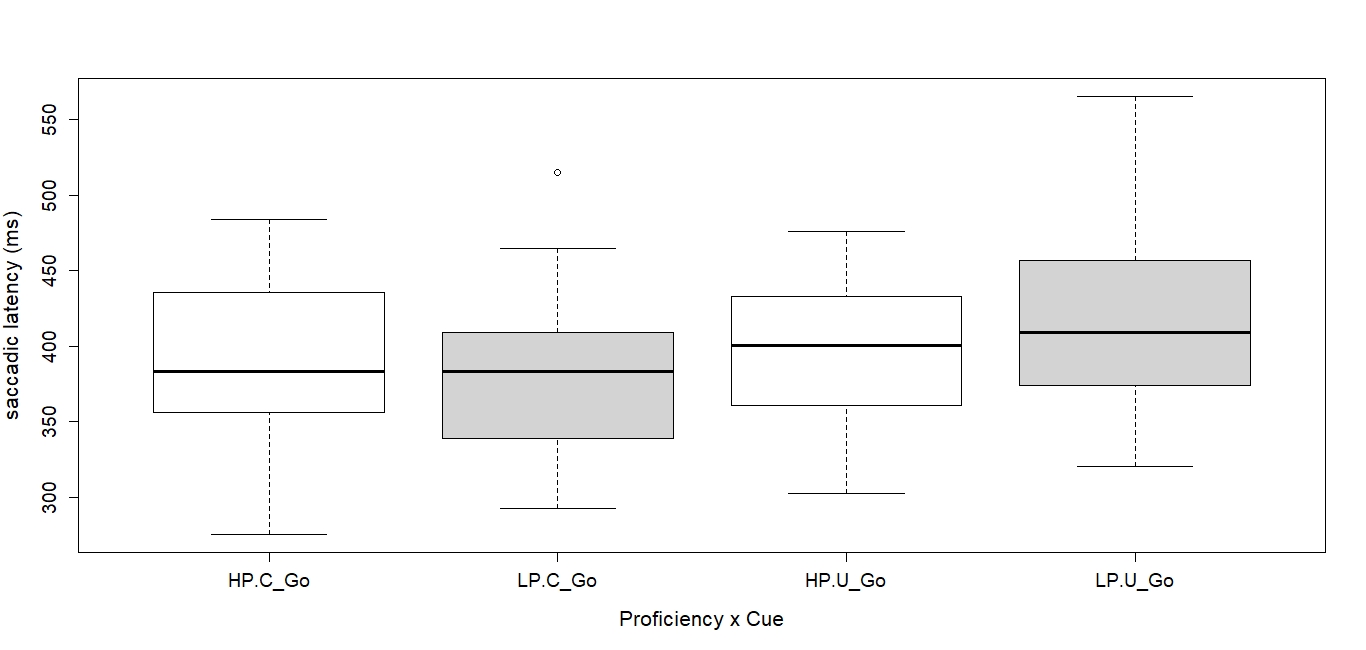


Figure 1: Box plot for saccadic latency for cue type and proficiency. HP: High proficiency; LP: Low Proficiency; C_Go: certain go trial; U_Go: Uncertain go trial

**Inhibitory cost:**

We performed a linear mixed effects analysis to look at the relationship between proactive inhibitory cost and second language proficiency. As fixed effects, proficiency (low, high) was entered into the model. As random effects, we had by item (stimulus: target living and non-living objects) random intercepts and by item random slope for the effect of proficiency on inhibitory cost. Visual inspection of residual plots did not reveal any obvious deviations from normality. The *p*-values were obtained by likelihood ratio tests of the full model with the effect in question (Proficiency) against the base model without the effect in question.

Model 2 <- lmer(cost ~ Group + (Group | Item) ,data, REML=FALSE)

Table 2. Linear mixed effect models for comparing the proactive inhibitory cost of high- and low- proficiency bilinguals

Random effects:

Groups Name Variance Std.Dev. Corr

Item (Intercept) 0.0 0.00

GroupLP 2.383-13 4.88e-07 NaN

Residual 733.8 27.09

Fixed effects:

Estimate Std. Error t value

(Intercept) 9.887 5.119 1.931

GroupLP 26.868 7.623 3.525

*Note*. LP: Low proficiency

Results showed that low proficiency bilinguals had greater proactive inhibitory cost by about 26.86 ms compared to the high proficiency group with a lower intercept value. With proficiency as a fixed factor variability due to random factor is near to zero and is mostly due to unexplained factors.

Statistics suggests that second language proficiency affects proactive inhibitory cost (χ^2^(3) = 11.11, *p*=0.01), increasing it by about 26.86 ± 7.62 (standard errors) for low proficient bilinguals.


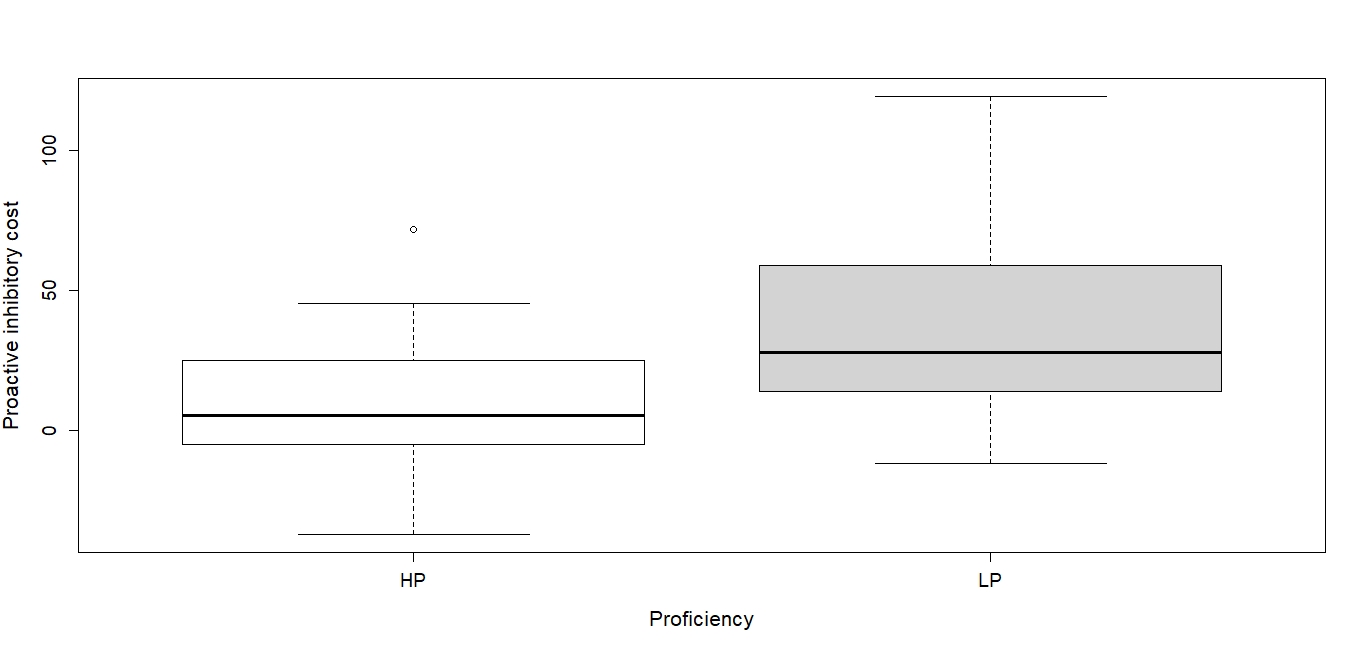


Figure 2: Box plot for proactive inhibitory cost for high and low proficient group. HP: High proficiency; LP: Low Proficiency.

**Previous trials analysis:**

We performed a linear mixed effects analysis to look at the relationship between second language proficiency and proactive adjustments in control. As fixed effects, we entered proficiency (low, high), previous trials (certain go, uncertain go, uncertain nogo) and current trial (certain go, uncertain go) (with interaction term) into the model. As random effects, we had by subject and by item (stimulus: target living and non-living objects) random intercepts and by item random slope for the interaction between proficiency, present trial type and previous trial type. Visual inspection of residual plots did not reveal any obvious deviations from normality. The *p*-values were obtained by likelihood ratio tests of the full model with the effect in question against the model without the effect in question.

Table 3. Linear mixed effect models for comparing the saccadic latencies of high- and low- proficiency bilinguals as a function of previous trial effects on the cued go/no-go task

Model3 <- lmer(RT ~ Group + present + previous + (present*previous) + (Group* present*previous) + (1 | ID) + (present * previous | Item) ,data, REML=FALSE)

Random effects:

Groups Name Variance Std.Dev. Corr

Subject (Intercept) 2922.44 54.06

Item (Intercept) 18.60 4.31

presentUG 7.44 2.73 1.00

previousUG 12.98 3.60 -1.00 -1.00

previousUN 37.04 6.08 1.00 1.00 -1.00

presentUG: previousUG 0.13 0.36 1.00 1.00 -1.00 1.00

presentUG: previousUN 44.04 6.36 -1.00 -1.00 1.00 -1.00 -1.00

Residual 2402.83 49.01

Fixed effects:

Estimate Std. Error t value

(Intercept) 387.62 14.13 27.42

GroupLP -10.92 20.53 -0.53

Current_UG 21.51 13.24 1.62

PreviousUG 5.08 13.35 0.38

previousUN 70.95 13.81 5.14

GroupLP:presentUG 24.68 19.50 1.27

GroupLP:previousUG 6.27 19.50 0.32

GroupLP:previousUN -8.65 19.51 -0.44

CurrentUG:previousUG -7.64 18.52 -0.41

CurrentUG:previousUN -23.76 19.127 -1.242

GroupLP:presentUG:previousUG -16.03 27.59 -0.58

GroupLP:presentUG:previousUN 47.09 27.59 1.70

*Note*. LP: Low-proficiency; UG: Uncertain Go; UN: Uncertain No-go.

Results showed overall slower saccadic latencies for current uncertain go trials compared to current certain go trials by about 21.51ms. The saccadic latencies were found to be slower for previous uncertain No-Go trials compared to previous certain go trials by about 70.94. The value of intercept was high (387.62). Difference between current uncertain Go trial and current certain Go trial is increasing for low proficient bilinguals, which suggests the effect of proficiency on current trial. Three-way interaction suggests that there is increment in saccadic latency difference (47.09) between high and low proficient bilinguals for the current uncertain Go trials when previous trial is uncertain No-Go in comparison to the previous certain go trial.

Saccadic latency varied as a function of previous and current trial types (χ^2^(30) = 127.2, *p*=0.001), increasing the latencies on the current uncertain go trial by about 47.09 ± 27.59 (standard errors) when the previous trial was an uncertain nogo trial in comparison to the the previous certain go trial for low proficient bilinguals. In general, saccadic latencies increased by 70.94 ± 13.80 when previous trial was an uncertain No-Go trial in comparison to the previous certain Go trial.


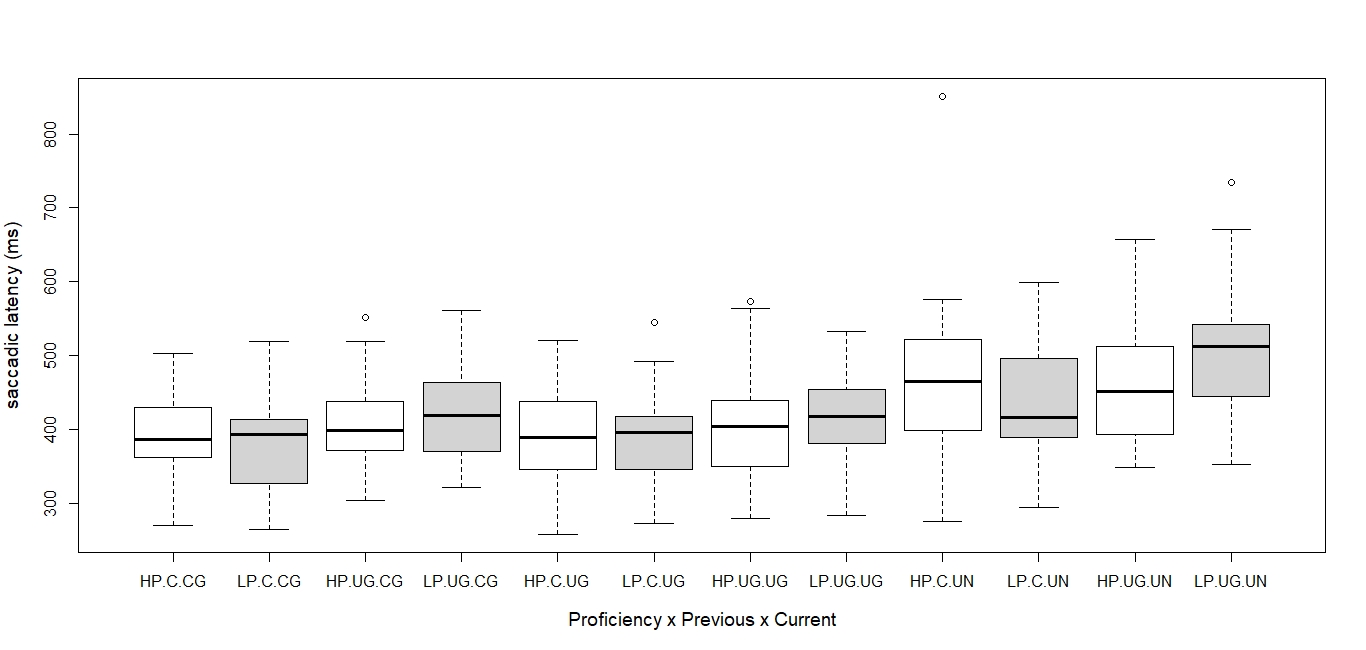


Figure 3: Boxplot for saccadic latency for both groups across previous and current trial types.

*Note*. HP: High proficiency; LP: Low-proficiency; C: previous certain go trial; CG: current certain go trial; UG: uncertain go; UN: uncertain no-go

**Experiment 2**

**Proficiency and CTOA**

We performed the linear mixed effects analysis to look at the relationship between saccadic latency and second language proficiency as a function of varying CTOAs. As fixed effects, we entered proficiency (low, high) and CTOAs (100, 300, & 500) into the model. As random effects, by subject and by-item intercepts and by item slope were entered into the model for the interaction between proficiency and CTOAs. Visual inspection of residual plots did not reveal any obvious deviations from normality. The *p*-values were obtained by likelihood ratio tests of the full model with the effect in question against the model without the effect in question.

Table 4. Linear mixed effect models for comparing the saccadic latencies of high- and low- proficiency bilinguals as a function of CTOAs on a cued go/no-go task

Model4 <- lmer(RT ~ Group + CTOA + (Group*CTOA) + (1 | Sub) + (Group + CTOA | Item) ,data, REML=FALSE)

Random effects:

Groups Name Variance Std.Dev. Corr

Subject (Intercept) 2446 49.46

Item (Intercept) 0.00 0.00

GroupLP 4.02e-11 6.34e-06 NaN

CTOA300 3.21e-11 5.67e-06 NaN -0.87

CTOA500 6.92e-11 8.32e-06 NaN -0.93 0.64

Residual 1105 33.24

Fixed effects:

Estimate Std. Error t value

(Intercept) 281.0917 11.0657 25.402

GroupLP -14.7655 16.4441 -0.898

CTOA300 -0.9683 8.7287 -0.111

CTOA500 -3.0800 8.7287 -0.353

GroupLP:CTOAC300 38.1649 12.9712 2.942

GroupLP:CTOAC500 35.8388 12.9712 2.763

Difference (38.16) between CTOA100 and CTOA300 condition is different when comparing low and high proficient bilinguals. Difference (35.83) between CTOA100 and CTOA500 condition is different when comparing high and low proficient bilinguals.

Difference between group (14.76), CTOAC300 (0.96), and CTOAC500 (3.08) is low as value of intercept is high (281.09). The intercept value is high by about 281.09.With proficiency and CTOA as a fixed factor, variability due to random factor is high for subject and lesser for item in comparison to subject and unexplained factors, variability due to unexplained variable is lesser than subject.

Second language proficiency affected saccadic latency (χ^2^(14) = 83.38, *p*=0.001), increasing it by about 38.16 ± 12.97 in CTOA300 and also for CTOA500 condition about 35.83 ± 12.97 for low proficient group.


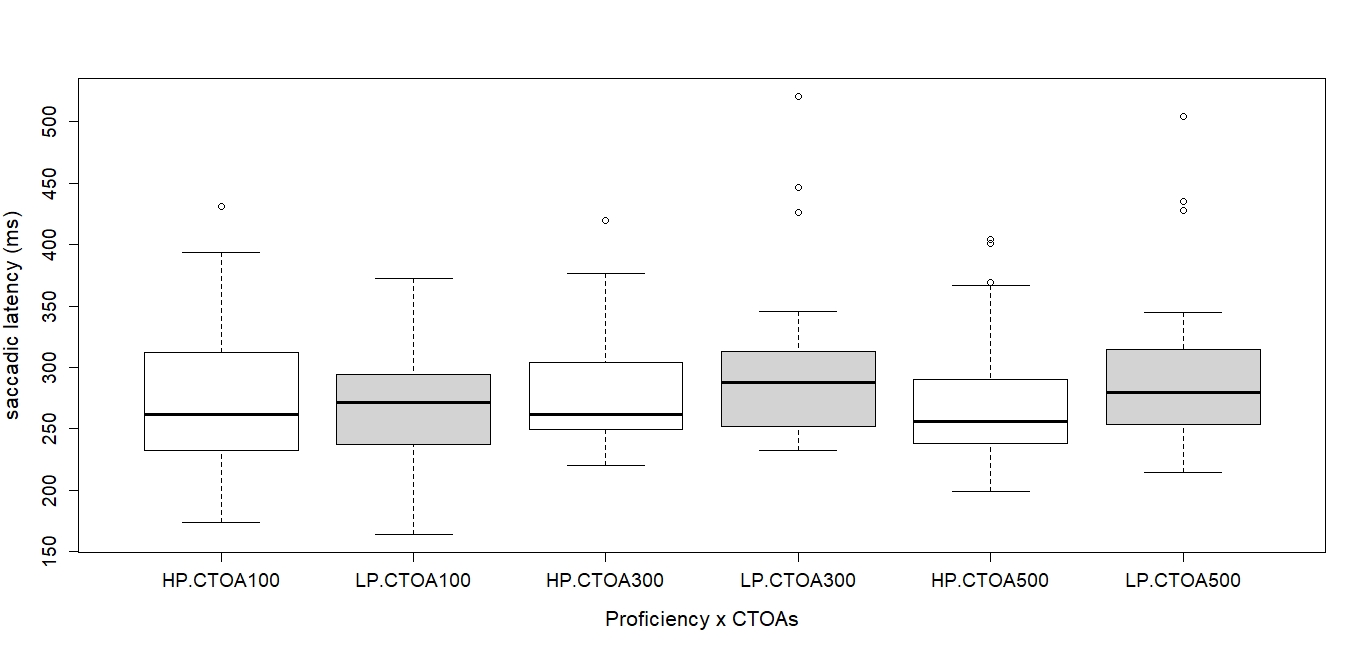


Figure 4: Boxplot for saccadic latency across CTOAs for high and low proficient group

*Note.* HP: High proficiency; LP: Low proficiency, CTOA: Cue to target onset asynchrony

**Proficiency and trial type**

We performed a linear mixed effects analysis to look at the relationship between saccadic latency for two conditions (CTOA500, white_cross_no_cue) and second language proficiency. As fixed effects, we entered proficiency (low, high), CTOA500 and white_cross_no_cue into the model. As random effects, by subject and by item (stimulus: target ‘X’ with cue type) intercepts and by-item slope were entered in the model for the interaction between proficiency and trial type. Visual inspection of residual plots did not reveal any obvious deviations from homoscedasticity or normality. The *p*-values were obtained by likelihood ratio tests of the full model with the effect in question against the model without the effect in question.

Table 5. Linear mixed effect models for comparing the saccadic latencies of high- and low- proficiency bilinguals as a function of trial type (white_cross_no_cue and CTOA 500 as control conditions).

Model 5 <- lmer(RT ~ Group + trial_type + (Group*trial_type) + (1 | Sub) + (trial_type | Item),data, REML=FALSE)

Random effects:

Groups Name Variance Std.Dev.

Subject (Intercept) 1113 33.36

Item (Intercept) 0 0.00

GroupLP 4.27e-10 2.06e-05 NaN

Trial_type_white_cross_no_cue 2.06e-09 4.54e-05 NaN 0.82

GroupLP: trial_type_white_cross_no_cue 9.93e-09 9.97e-05 NaN -0.83

Residual 2895 53.81

Fixed effects:

Estimate Std. Error t value

(Intercept) 278.01 11.76 23.647

GroupLP 21.07 17.47 1.206

White_cross_no_cue 37.66 14.13 2.665

GroupLP: trial_typewhite_cross_no_cue -37.97 21.00 -1.808

Difference between group (21.07), white_cross_no_cue (37.66), and for white_cross_no_cue trial type for low proficient is high as the intercept value is high. Low proficient bilinguals showed overall slower saccadic latencies by about 21.07. White_cross_no_cue showed slower latencies by 37.66ms compared to CTOA500ms and low proficient group showed faster white_cross_no_cue condition compared to CTOA500ms. With proficiency and trial type as fixed factor, variability due to subject as random factor is less than unexplained variables (residual), and less variability due to item as random factor for Group and trial_type interaction factor.

Second language proficiency affected saccadic latency (χ^2^(11) = 6.12, *p*= 0.86), increasing it by about 21.07 ± 17.47 for the low proficient group, and also increasing the same for white_cross_no_cue condition by about 37.66 ± 14.13. Low proficient group showed a lower value for white_cross_no_cue by about -37.97 ± 21.00. Current model is not significant but model with only intercept is significant.


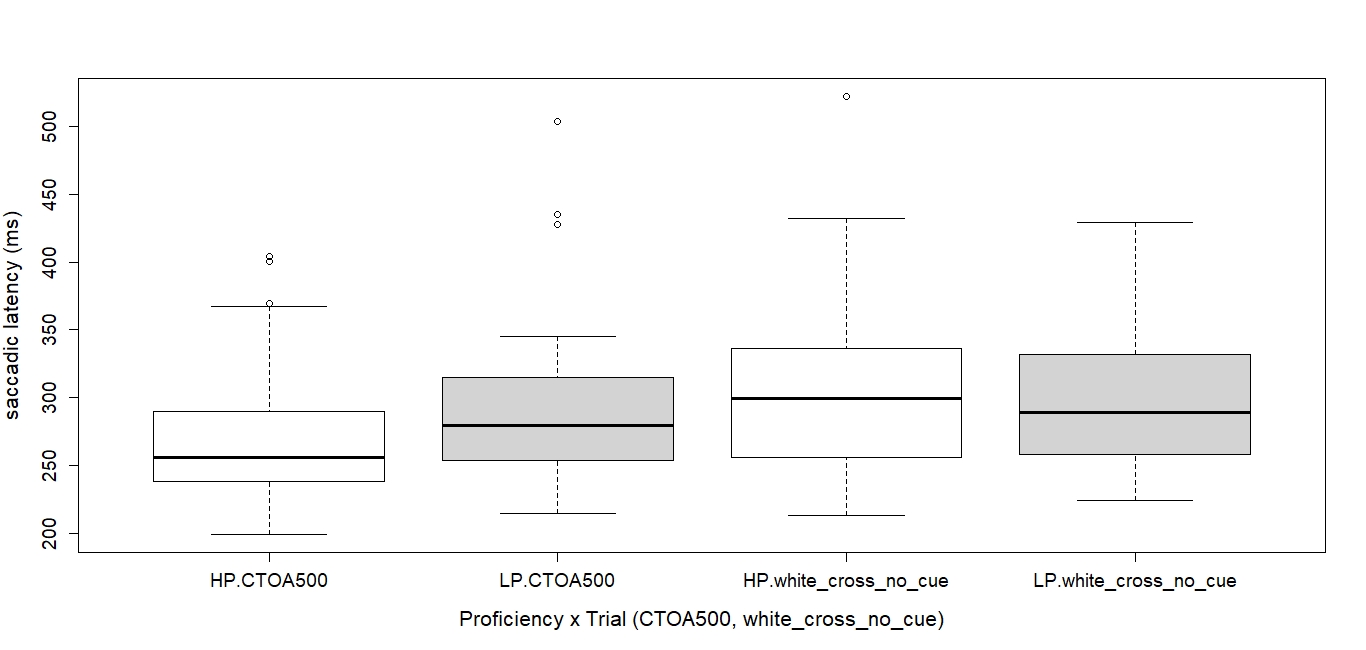


Figure 5: Boxplot for saccadic latency for high and low proficient group for CTOA500 and white_croos_no_cue trial type. *Note.* HP: High proficiency; LP: Low proficiency, CTOA: Cue to target onset asynchrony

**References**

1. R Development Core Team. R: A Language and Environment for Statistical Computing. Austria: R Foundation for Statistical Computing Vienna; 2011. ISBN 3-900051-07-0.
2. Bates D, Mächler M, Bolker B, Walker S. Fitting Linear Mixed-Effects Models Using lme4. J. Stat Softw. 2015; 67, 1-48.
3. Barr DJ, Levy R, Scheepers C, Tily HJ. Random effects structure for confirmatory hypothesis testing: keep it maximal. J. Mem. Lang. 2013; 68, 255–278. 10.1016/j.jml.2012.11.001
4. Jaeger, TF. Post to HLP/Jaeger lab blog. May 14 2009, http://hlplab.wordpress.com/2009/05/14/ random-effect-structure (cited in Barr et al., 2013)
